# Using Shakespeare's Sotto Voce to Determine True Identity From Text

**DOI:** 10.3389/fpsyg.2018.00289

**Published:** 2018-03-15

**Authors:** David Kernot, Terry Bossomaier, Roger Bradbury

**Affiliations:** ^1^National Security College, Australian National University, Canberra, ACT, Australia; ^2^National Security and ISR Division, Defence Science and Technology Group, Edinburgh, SA, Australia; ^3^The Centre for Research in Complex Systems, Charles Sturt University, Bathurst, NSW, Australia

**Keywords:** authorship identification, personality, sensory processing, principal component analysis, linear discriminant analysis

## Abstract

Little is known of the private life of William Shakespeare, but he is famous for his collection of plays and poems, even though many of the works attributed to him were published anonymously. Determining the identity of Shakespeare has fascinated scholars for 400 years, and four significant figures in English literary history have been suggested as likely alternatives to Shakespeare for some disputed works: Bacon, de Vere, Stanley, and Marlowe. A myriad of computational and statistical tools and techniques have been used to determine the true authorship of his works. Many of these techniques rely on basic statistical correlations, word counts, collocated word groups, or keyword density, but no one method has been decided on. We suggest that an alternative technique that uses word semantics to draw on personality can provide an accurate profile of a person. To test this claim, we analyse the works of Shakespeare, Christopher Marlowe, and Elizabeth Cary. We use Word Accumulation Curves, Hierarchical Clustering overlays, Principal Component Analysis, and Linear Discriminant Analysis techniques in combination with RPAS, a multi-faceted text analysis approach that draws on a writer's personality, or self to identify subtle characteristics within a person's writing style. Here we find that RPAS can separate the known authored works of Shakespeare from Marlowe and Cary. Further, it separates their contested works, works suspected of being written by others. While few authorship identification techniques identify self from the way a person writes, we demonstrate that these stylistic characteristics are as applicable 400 years ago as they are today and have the potential to be used within cyberspace for law enforcement purposes.

## Introduction

Little is documented about Gulielmus (William) Shaksper or Shakspere, the person, outside of his christening at Stratford-on-Avon on 26 April 1564 and his marriage to Ann Hathaway in November 1582, whom he had three children with: a daughter Susanna born in 1583, and twins, Hamnet and Judith, born in 1585 (Kreeger, 1987; Ellis, 2000). However, by 1623 and seven years after his death, more than 37 plays, at least four narrative poems, and 154 sonnets had been published in London. William Shake-speare, or Shakespeare, began to be identified as the author of these works, and over the next 200 years, this solidified into a tradition (Kreeger, 1987).

Benjamin Disraeli, Lord Beaconsfield, was the first to place doubt on William Shakespeare's identity in 1837, and since then the question of the authorship of Shakespeare's publications has engaged a wide range of prominent people (Krsul and Spafford, 1997).

This ongoing controversy has engaged many analysts. There are those that defend Shakespeare as the author, while others focus on authorship identification in general. We are in the latter group and believe this is a very fertile place to test new methods. This project is motivated by our interest in using these techniques to identify assailants in cyberspace for law enforcement purposes where authorship identification is critical (Kaminski, 2013; Kambourakis, 2014).

Although Edward de Vere, the Seventeenth Earl of Oxford has been named as a very strong candidate from a pool of 56 candidates, four significant figures in English literary history, Bacon, de Vere, Stanley, and Marlowe, are thought to be the most likely alternatives to Shakespeare (Kreeger, 1987).

In 1901, Mendenhall counted the length of words and used word-length frequency distributions to separate the authored plays of William Shakespeare from Francis Bacon, and a further study highlighted that the word-length distribution of Christopher Marlowe's plays was more aligned with Shakespeare's style (Tuldava, 2004).

Elliot and Valenza (1991) used a different identification technique and conducted modal testing based on word usage to highlight the different style of Shakespeare's poems to those of Edward de Vere and suggested that de Vere was not the author of the Shakespeare work.

Little is known of the creative poems of Ferdinando Stanley, also known as Lord Strange and the Fifth Earl of Derby, but he was likely associated with Shakespeare through his company of actors (May, 1972). Many believe that Shakespeare was a member of Ferdinando's acting company in the early 1590s, known then as Lord Strange's Men, before the next in line to the throne was assassinated in 1594 (Daugherty and Press, 2011).

In 1920 doubt was raised about the authorship of the play *Titus Andronicus*, suggesting it was a pre-Shakespearian play, and retouched by Shakespeare while it was in possession of Lord Strange's men (Gray, 1920). Around the same time, Marlowe's involvement in Shakespeare's *Henry VI* was also suggested (Brooke, 1922), and today, there is still uncertainty about the influence and collaboration between Shakespeare and Marlowe (Merriam, 1998).

Other scholars have applied different techniques to the Shakespeare identification problem. Matthews and Merriam (1993) used a neural computational pattern recognition technique on Shakespeare and Fletcher with considerable reliability. They extended their technique to the works of Shakespeare and Marlowe (Matthews and Merriam, 1993). Thirty-six Shakespeare plays, and seven Marlowe were tested. Using 10 canonical plays from Shakespeare and three of Marlowe's plays, Merriam and Matthews (1994) trained their model using 51 thousand word samples before subsequently classifying the remaining 26 complete plays of the Shakespeare First Folio and the remaining four from Marlowe. They successfully classified 93% of the plays. They used five discriminants that comprised of a series of ratios using different combinations of the following 14 function words: *but, by, did, do, for, no, not, on, so, that, the, to, upon*, and *with*.

In the last decade, the interest in the Elizabethan playwrights has not faded. Recent work on Marlowe and Shakespeare by Tearle et al. (2008) highlight that Shakespeare was a collaborator on Titus Andronicus, but that it was easy to separate Shakespeare from Marlowe using neural networks. Craig and Kinney (2009) suggest that there is doubt about the authorship of Henry VI and that Parts 1 and 2 are Marlowe's and not Shakespeare's. Zhao and Zobel (2007) suggest that Marlowe did not write the works of Shakespeare. Much of the recent findings are due to the processing power of the computer and some recent techniques.

Stylometric analysis, the quantitative analysis of a text's linguistic features, can be traced back to Augustus de Morgan's resolution of authorship disputes using the frequency of word lengths in 1851. The first manual quantitative analysis occurred in the late 1880s by Thomas C Mendenhall (1887) who used word length distributions from the works of Bacon, Marlowe, and Shakespeare to identify the authorship of Shakespeare's plays. Stylometry has been used extensively to determine the authorship of many undocumented playwright collaborations from the Elizabethan period, including Shakespeare (Segarra et al., 2017). Below we summarize some analytical techniques, but for a more comprehensive overview of stylometry and its classification techniques see Neal et al. (2017) and Aljumily (2015).

Many of the stylometric text analysis techniques rely on basic statistical correlations, word counts, collocated word groups, or keyword density (Matsuo and Ishizuka, 2004; Leech and Onwuegbuzie, 2007; Lamb et al., 2013). There are many different techniques in use today on Shakespeare and others, from n-grams (Frantzeskou et al., 2007), and Latent Semantic Analysis (Raju et al., 2016), to machine learning techniques (Jockers and Witten, 2010). However, there does not appear to be any single technique. Juola (2006) concludes that the best choice of the feature set is strongly dependent upon the data analyzed and no method has yet emerged as being particularly good. Rudman (2012) revisited the problem, 13 years after his earlier critique (Rudman, 1998) and after well over a further 600 studies concluded there is still no consensus as to the correct methodology or technique for authorship attribution.

There appears dissension among leading Shakespearean authorship attribution scholars about an agreed method (Rudman, 2016), but the most successful and robust methods rely on low-level information such as character n-grams or auxiliary word (function words and stop words such as articles and prepositions) frequencies (Stamatatos, 2009). The premier work in evaluating authorship in the 16th to mid-17th centuries includes MacDonald P. Jackson, Brian Vickers, and Hugh Craig (Segarra et al., 2017). Jackson (2006) uses common low-frequency word phrases, repetition of phrases, collocation, and images to link word groups to other works. Vickers (2011) uses a tri-gram, or n-gram, approach, while (Hirsch and Craig, 2014) use function word frequency. They also use methods based on the Information Theoretic measure Jensen-Shannon divergence (JSD), and unsupervised graph partitioning clustering algorithms (Arefin et al., 2014). There are other techniques used in this period of Shakespearean analysis, including simple function words (Matthews and Merriam, 1993; Merriam and Matthews, 1994) and word adjacency networks (WANs) (Segarra et al., 2017), or looking at rare and unique phrases (Swaim, 2017). However, the most relevant to the RPAS technique used in this paper are the ones based on personality. The meaning-extracting method (MEM) from the field of psychology (Chung and Pennebaker, 2008; Boyd and Pennebaker, 2015) is used to extract themes from commonly used adjectives and describe a person from their personality. Pennebaker et al. (2015), Litvinova et al. (2016) and Skillicorn et al. (2017) are developing personality aspects of human language to improve authorship profiling. The ability to profile user personality and infer stable differences in individual behavior from writing can be used to predict a person's preferences and future behavior with sufficient accuracy (Wright and Chin, 2014).

In this paper, the authors offer a new and alternative approach to authorship identification using personality. We attempt to get better clarification by going beyond statistics and blind classification and attempt to infer a person's personality: their sense of self. It can be found in the subtle characteristics hidden in a person's writing style (Northoff et al., 2006; Argamon et al., 2009; Iqbal et al., 2013). Voice is the manifestation of author's will, intent, and feeling, and it is the animus of storytelling (Charmaz and Mitchell, 1996). The authorial voice projects an image of the author (Lorés-Sanz, 2011), and we think of this as “sotto voce”, the voice of the author that can't help but utter an involuntary truth about their identity.

Others claim to see Shakespeare's voice within his narrative. Klein (1993) says it is apparent in the guise of Hamlet's father and bound intrinsically to Shakespeare's creation. It appears in the poem, *The Phoenix and the Turtle*, as a three-part structure that foregrounds Shakespeare's voice (Cheney, 2009). It is also evident in the voice of the speaker in The Sonnets (Kambasković-Sawers, 2007), where “Shakespeare the man” can be reconstructed more completely here than from any of his other works (Burnham, 1990). We suggest that this voice, a person's sense of self, is reflected throughout all the works of Shakespeare, Marlowe, and Cary, and is an example of sotto voce. It can be used to determine an author's true identity.

Some of the techniques used here are not new. Richness is not, and Mendenhall used word frequency charts to separate the writings of different authors (Mendenhall, 1887). Using function words to reveal personality traits is recent but also not new (Pennebaker, 2011). Principal Component Analysis (PCA) has been used extensively since the 1980's to separate the authorial styles of Shakespeare and other Elizabethan playwrights (Burrows and Craig, 2012).

However, we apply these reliable techniques to the Elizabethan playwrights to highlight the consistency of our results against other well-documented results. The creation of a stylistic fingerprint of a person from a combination of a person's internal gender, their use of sensory-based adjectives factored across the five sensory modalities, and using specific function words that have high levels of concreteness and imagery scores which reflect self, or sotto voce is new. We further highlight, how depressed a person may be from their writing. While outside the scope of this study, it is part of a broader body of work that is looking at using these techniques, particularly within the law enforcement area, where depression and the cognitive state of an individual's mental state is a valuable identifier. Using techniques that draw on biomarkers for creativity and a person's known psychological state (Rosenstein et al., 2015; Zabelina et al., 2015), we identify characteristics of William Shakespeare, Christopher Marlowe, and Elizabeth Cary that allow us to separate their work using a new technique RPAS.

## Material and methods

### Preparing the text

The works of William Shakespeare's are sourced from the Massachusetts Institute of Technology's (MIT, 1993) the Complete Works of William Shakespeare, and Christopher Marlowe from Farey (2014). We also process the 1613 play, *The Tragedy of Mariam, the Fair Queen of Jewry* by English poet and dramatist, Elizabeth Cary (Mark, 2014), published when Shakespeare ceased writing. This ensures an independent female writer for use in some tests. These versions use Modern English spelling but still contain Early Modern English words where they cannot be directly transcribed, (such as ‘tis!; thou; doth, fix’d; o'er) and included for consistent word richness scores.

The Complete Works of William Shakespeare has been online since 1993 as the Complete Moby(tm) Shakespeare. It stemmed from the Globe Shakespeare, a mid-nineteenth-century popular edition of the [old] Cambridge Shakespeare, and based on Shakespeare's First Folio published in 1623, although more than half of the 36 plays come from earlier editions in quarto. There are substantial textual differences between even the earliest surviving copies of Shakespeare's plays, and these copies are the result of an editorial process.

We divide William Shakespeare's histories, comedies, tragedies, poems and sonnets, Christopher Marlowe's plays and poems, and Elizabeth Cary's play into 57 pseudo-random textual chunks, or files. Each time we encounter a title heading in each work, we create a new file (Table 1). This means that some chunks are partial works, such as *The Passionate Pilgrim* (chunks 23-25, and 41), *The Phoenix and the Turtle* (chunks 29-30) and *The Passionate Shepherd to His Love* (chunks 55-56). Theatrical stage direction is removed from the text (speaker titles, play actions and lists of characters for each scene) and we process the files with the Stanford Parts Of Speech Tagger (Toutanova and Manning, 2000) to easily group and remove punctuation. While the tagger uses the Penn Treebank labels based on today's linguistic structure, these influences can be ignored because any variations are applied consistently across the dataset, and further they do not impact on the RPAS approach. Rather than remove the stop words—extremely common words—as is standard practice, our method uses these prepositions and article word types, and we only remove punctuation and symbols. The word corpus is aggregated by frequency for each chunk. We analyse the corpus parts-of-speech tags to ensure it shows no biases and we construct a multi-dimensional vector from the results of applying the RPAS technique. While studies have successfully been conducted on one or two authors and with a single word group containing as few as 14 different words (Matthews and Merriam, 1993), this study follows a newer approach using larger datasets (Taylor and Egan, 2017). It has three authors' works across a corpus of 1.031 million words and uses 507 different words (see Table S2 External Data). This multivariate approach also applies novel psycholinguistic and modal weightings as described below.

**Table 1:** Shakespeare, Marlowe, and Cary's Works and how they were broken into chunks.

**ID**	Year^*^	**Title**	**Type**	**Short title**	**In work**
**WILLIAM SHAKESPEARE**					
1	1589	Comedy of Errors	C	C1	Comedy of Errors
2	1590	Henry VI, Part II	H	H1	Henry VI, Part II
3	1590	Henry VI, Part III	H	H2	Henry VI, Part III
4	1591	Henry VI, Part I	H	H3	Henry VI, Part I
5	1592	Richard III	H	H4	Richard III
6	1593	Taming of the Shrew	C	C2	Taming of the Shrew
7	1593	Titus Andronicus	T	T1	Titus Andronicus
8	1593	Venus and Adonis	P	P1	Venus and Adonis
9	1594	Love's Labour's Lost	C	C4	Love's Labour's Lost
10	1594	Romeo and Juliet	T	T2	Romeo and Juliet
11	1594	The Rape of Lucrece	P	P2	The Rape of Lucrece
12	1594	Two Gentlemen of Verona	C	C3	Two Gentlemen of Verona
13	1595	Midsummer Night's Dream	C	C5	Midsummer Night's Dream
14	1595	Richard II	H	H5	Richard II
15	1596	King John	H	H6	King John
16	1596	Merchant of Venice	C	C6	Merchant of Venice
17	1597	Henry IV, Part I	H	H7	Henry IV, Part I
18	1597	Henry IV, Part II	H	H8	Henry IV, Part II
19	1598	Henry V	H	H9	Henry V
20	1598	Much Ado about Nothing	C	C7	Much Ado about Nothing
21	1599	As You Like It	C	C9	As You Like It
22	1599	Julius Caesar	T	T3	Julius Caesar
23	1599	Love's Answer	P	P5	The Passionate Pilgrim
24	1599	Sonnets to sundry notes of music	P	P4	The Passionate Pilgrim
25	1599	The Passionate Pilgrim	P	P3	The Passionate Pilgrim
26	1599	Twelfth Night	C	C8	Twelfth Night
27	1600	Hamlet	T	T4	Hamlet
28	1600	Merry Wives of Windsor	C	C10	Merry Wives of Windsor
29	1601	The Phoenix and the Turtle	P	P6	The Phoenix and the Turtle
30	1601	Threnos	P	P7	The Phoenix and the Turtle
31	1601	Troilus and Cressida	C	C11	Troilus and Cressida
32	1602	All's Well That Ends Well	C	C12	All's Well That Ends Well
33	1604	Measure for Measure	C	C13	Measure for Measure
34	1604	Othello	T	T5	Othello
35	1605	King Lear	T	T6	King Lear
36	1605	Macbeth	T	T7	Macbeth
37	1606	Anthony and Cleopatra	T	T10	Anthony and Cleopatra
38	1607	Coriolanus	T	T8	Coriolanus
39	1607	Timon of Athens	T	T9	Timon of Athens
40	1608	Pericles	C	C14	Pericles
41	1609	A Lover's Complaint	P	P8	The Passionate Pilgrim
42	1609	Cymbeline	C	C15	Cymbeline
43	1609	Sonnets	P	P9	Sonnets
44	1610	Winter's Tale	C	C16	Winter's Tale
45	1611	Tempest	C	C17	Tempest
46	1612	Henry VIII	H	H10	Henry VIII
**CHRISTOPHER MARLOWE**					
47	1590	Tamburlaine Part I		M1	Tamburlaine The Great Part I
48	1590	Tamburlaine Part II		M2	Tamburlaine The Great Part II
49		Edward II	H	M3	Edward II
50		The Jew of Malta	T	M4	The Jew of Malta
51		Doctor Faustus		M5	Doctor Faustus
52		Dido Queen of Carthage		M6	Dido Queen of Carthage
53		The Massacre at Paris		M7	The Massacre at Paris with the Death of the Duke of Guise
54		Hero and Leander	P	M8	Hero and Leander
55		The Passionate Shepherd	P	M9	The Passionate Shepherd to His Love
56		Walter Raleigh	P	M10	The Passionate Shepherd to His Love
ELIZABETH CARY					
57	1612	The Tragedy of Mariam	T	EC1	The Tragedy of Mariam, the Fair Queen of Jewry

*Type: C, Comedies; H, Histories; T, Tragedies; P, Poems*.

* *The Year may not have any bearing as many works may well have been written earlier. In Marlowe's case, all but two of his works were published after his death*.

### The RPAS method

#### Richness (R)

Richness (Equation 1) is a measure of a person's ability to use a vocabulary of a determined size and based on Menhinick's (1964) species diversity equation. It is the number of unique words used by an author and linked to education and age (Hartshorne and Germine, 2015). It is not a measure of all of the words in the English language. While the average English speaker has a passive vocabulary of about 100,000 words (Pennebaker, 2011), we are interested in Shakespeare's active vocabulary, hence limit the document size to around 30,000 words, the size of the largest Shakespeare work, rather than using smaller chunks and averaging. The Richness score can be determined by:

Equation 1: Richness

$$\begin{array}{lll} Richness\;\left(R\right) & = & \frac{w}{N} \end{array}$$

Where *w* = number of unique words or types in the document, and *N* = total document word count or tokens.

There are theoretical limits to this equation, and the size of documents must be carefully controlled to avoid artifacts. Eventually, the value will reach an asymptote when no new words are found. Near that point, the larger the document size, the smaller the Richness score will be (0 *as N* → ∞).

The type-token ratio (TTR) can be considered a variant of Menhinick's (1964) species diversity equation that measures vocabulary richness. TTR is one of the oldest and easiest ways of measuring richness but it is dependent on text size, and while many attempts to reduce this problem have been proposed no one has been fully successful (Kubát and Milička, 2013). The biggest criticism of TTR is that it should not be used on its own, rather it should be incorporated into a larger suite of techniques (Vermeer, 2000; Kubát and Milička, 2013). We avoid this by using the RPAS multivariate technique.

#### Personal pronouns (P)

A person's personal pronouns use (Equation 2 or see Kernot, 2013 for further detail) provides a score that can identify an author's unique style on a continuum between 0 and 1 and can differentiate between authors of the same or different sex. The formula draws from the binary logistic regression, also called a logit model, where a series of regression coefficients represent the change in the criterion for each predictor. In this case, we draw on two existing studies on gender (Argamon et al., 2003; Kernot, 2013) and use the equation based on the three best predictors of a person's socially constructed gender (Cheng et al., 2011). The Argamon et al. (2003) study analyzed 25 million words in 604 documents using a range of fiction and non-fiction articles (natural science, applied science, social science, world affairs, commerce, arts, belief/thought, and leisure) from the British National Corpus to assign a dominant gender across 29 statistically significant personal pronouns. These results were further distilled (Kernot, 2016) and statistically significant gender identities determined to 90% accuracy using three personal pronouns from a collection of 25 thousand words, using articles from the internet (news reports, web articles, personal blog posts, book extracts, and an oration).

Gender can also be expressed as a Masculine (M) or Feminine (F) style. Where the Personal pronouns score is greater than or equal to 0.5, we would allocate an M categorical value. The Personal pronouns score can be determined by:

Equation 2: Personal pronouns

$$\begin{array}{l} personal\;pronouns\;\left(P\right)\\ =\;\frac{\exp\left(-0.93-451.86\alpha +322.47\beta +129.83\gamma \right)}{1+\text{exp}\left(-0.93-451.86\alpha +322.47\beta +129.83\gamma \right)} \end{array}$$

*Where: Masculine style* (*P*) ≥ 0.5, and Feminine style (*P*) < 0.5 And α = ‘My’, β = ‘Her’, and γ = ‘Its’

It should be noted that Shakespeare's Early Modern English is much closer to today's language than that of Old or Middle English and most personal pronouns have maintained number, case, and gender throughout the history of English (Horobin, 2010). However, its only came into print in 1598, and *his* was a neuter possessive where today we would use *its*, noting that Shakespeare's First Folio, printed in 1623, kept the earlier form of *his* (Nevalainen, 2006). While we could replace *its* with *his*, there are 13 of Shakespeare's works that contain the word *its*, and we elect not to replace *his* for *its*. This approach does not affect the algorithm's effectiveness in comparing data from within the Early Modern English period. Replacing *its* with *his* would change the gender category of two poems, however, and we will mention that later.

#### Referential activity power (A)

Grounded in “Critical Realism,” the American philosopher, Roy Wood Sellars (Sellars, 1959), provided a linguistic framework guided by the brain's sensory referential sensations and that concept was picked up for clinical studies into depression (Bucci and Freedman, 1981; Bucci, 1982, 1984; Bucci and Miller, 1993).

Clinical psychologists use Referential Activity (RA) to score a person's level of depression from their speech. This occurs across the following four categories: properties of actual things or events or to anything that is experienced as a sensation or feeling sensory characteristics of language (Concreteness); the vividness and effectiveness of language in reflecting and capturing imagery or emotional experience, in any sense modality (Imagery), and; the degrees of articulation, focus and communicative style (Specificity and Clarity) (Bucci and Kabasakalian-McKay, 2004). While the RA measure assesses the degree to which a speaker or writer can translate experiences into words, we believe it can map a continuum of a cognitive state from a healthy individual to one who has been diagnosed with depression.

A person's personality, their sense of self can be measured in terms of their use of a group of function words known as particles, and include pronouns, articles, prepositions, conjunctives, and auxiliary verbs, and they can serve as markers of emotional state, social identity, and cognitive styles to capture the ability to verbalize nonverbal experiences through Referential Activity (Pennebaker et al., 2003).

We focus on the sensory aspects of Bucci's concepts of Referential Activity and use two of the four categories, the sensory characteristics of language (Concreteness) and the effectiveness of language to capture imagery and emotional experience in any sensory modality (Imagery). We also draw on Pennebaker et al.'s idea that particles can reflect the sense of a person's self, and use the Medical Research Corporation (MRC) Psycholinguistic database (Coltheart, 1981). We select articles, conjunctives, prepositions, and pronouns that have concreteness and imageability scores greater than 0.

These 117 highly concrete and imageability function word scores have been averaged for each word and these scores, ε_*i*_ can be found in the External Data. We create four referential categories, one each for articles, conjunctives, prepositions, and pronouns.

If we let the number of words in each referential category, *i, be ω*_*i*_ *and ε*_*i*_, the weight for each category then the RA Power score, *A*_*k*_ (Equation 3) can be determined by:

Equation 3: Referential Activity Power

$$\begin{array}{l} RA\;Power\;\left(A_{k\;1-4}\right)=\overset{N_{k}}{\sum }\frac{\omega_{i}^{2}\varepsilon_{i}}{D} \end{array}$$

Where ∑ *N*_*k*_ = 117, and D is the number of words in the document.

The data is normalized based on the document or chunk size so that the ratio of richness to Referential Activity Power is independent of document size. While word counts are squared to emphasize the difference in the range of values, we ignore the effects of power and focus on the way the variables capture the variance in the number of words used that are then multiplied by the RA category weight, ε_*i*_ across the different works (see Table S3 External Data).

#### Sensory adjectives (S)

Many Sensory (S) words are processed by the brain as sight/feel and smell/taste word categories (Lynott and Connell, 2009 For more information see Miller, 1995; Kernot, 2013; Fernandino et al., 2015). We use adjectives over verbs or nouns because they appear more frequently in text and their context is not necessary. We draw on a study of 387 adjectives (van Dantzig et al., 2011). These have been analyzed in two different contexts to assess the dominant visual (V), auditory (A), haptic (H), olfactory (O), or gustatory (G) sensory modality the word responds to. The study provides a list of 774 words because they were each tested in the two most dominant modalities. These 774 sensory words are allocated an exclusivity score, φ_*i*_ (found in the External Data) that reflects the brain's Representational System. We believe it can be used to capture the sensory gating biomarker characteristics of a person that in turn can construct a signature of a person's unique sensory cortex functions.

There are five sensory categories, one each for V, A, H, O, G. If we let the number of words in each sensory category, *i, be φ*_*i*_ *and ϑ*_*i*_, the weight, or exclusivity score for each category then the Sensory Adjectives, Sk (Equation 4) can be determined by:

Equation 4: Sensory score

$$\begin{array}{l} Sensory\;adjectives\;\left(S_{k\;1-5}\right)=\overset{N_{k}}{\sum }\frac{\varphi_{i}\vartheta_{i}}{D} \end{array}$$

Where ∑ *N*_*k*_ = 774, and D is the number of words in the document (see Table S4 External Data).

### Correlation analysis

We use the Statistical Package for the Social Sciences (SPSS), and test the independence of the RPAS variables in the data and measure the degree of correspondence between the variables with the Pearson Product Moment Correlation or “r” (Burns and Burns, 2008). We run three tests. In the first, we test the independence of the four high-level elements, Richness (R), personal pronouns (P), Referential Activity Power (A), and Sensory Adjectives (S). We test the sensory adjectives that make up the Sensory VAHOG elements: V–visual; A–auditory; H–haptic, O–olfactory, and G–gustatory. We also test the four linguistic variables known as particles that make up Referential Activity Power: A–articles; C–conjunctives; P–prepositions; and PRON–pronouns. We interpret the correlation size using Burns and Burns (2008:346) descriptions.

### Word accumulation cures

There are theoretical limits to Menhinick's Index used to measure species diversity or species richness that we use above in section Richness (R) to describe Richness (R). Eventually, the value will reach the total species richness asymptote as no new species are found (Walther and Morand, 1998). In ecology, the size of the area searched impacts on the possible sample size because it is the number of species collected in a particular area and not every possible sample that exists and the measurement is species density (James and Wamer, 1982). The species accumulation curve is an intuitive way to compare the richness of two samples of different sizes (Gotelli and Colwell, 2011). The species discovery curve or species accumulation curve is linked to empirical Zipf distributions and can highlight differences in word frequency distribution (Bentz et al., 2014).

From Ecology, we can create a graph where the x-axis records the number of individuals sampled, and the y-axis records the cumulative number of species recorded. Regardless of the species abundance distribution that is plotted as a result of this graph, the curve increases monotonically, with a decelerating slope (Gotelli and Colwell, 2011). We can also use this to plot word distribution, where the x-axis can be the document sample size and the y-axis can be the number of unique words. The curve will respond the same way.

This type of curve that plots word frequency can be used to estimate the total vocabulary of a writer from a given sample (Efron and Thisted, 1976). We create two charts to examine Richness: an Accumulative Word Type Usage Curve for the largest 100 word types, and a Word Accumulative Curve.

An Accumulative Word Type Usage Curve for the largest 100 word types is calculated so that we can examine the Richness of the Shakespeare and Marlowe corpus from their plotted curves using the example in Efron and Thisted (1976). Initially, we create a word type frequency list of the Shakespeare corpus and order the data from the smallest number of unique words (types) to the largest. We aggregate the data for the first 100 word groups. We do the same to the smaller Marlowe data and plot the results of both playwrights. The number of word groups (largest 100) appears on the x-axis, while the number of accumulated unique word types appear on the y-axis. We then visually compare the asymptotes of both playwrights using a different Word Accumulative Curve from the one mentioned in the previous paragraph. In this one, each of the works of Shakespeare are ordered from the largest work size (number of individual tokens) to the smallest. Then the number of unique words in each work (new types) introduced is calculated. This data is then aggregated, and we have a data point for each file that introduces new unique words (types). This process is applied to the works of Marlowe. We plot both playwrights. The accumulated words are written in thousands (document sample size/number of tokens) appears on the x-axis, while the accumulated unique words in thousands (number of unique words/types) appears on the y-axis.

The values of lexical richness change for different measures used because of text length, and it is necessary to correct for this (Tweedie and Baayen, 1998). We do this with ratios (Singhal et al., 1996; Kessler et al., 1997) because we are effectively examining the word density within each chunk and comparing it to the others (Gotelli and Colwell, 2011), and any global richness coefficient can, therefore, be ignored.

### Three complementary clustering techniques

The data is clustered using three complementary techniques. The first attempts to separate the playwrights, the second separates known works from contested works—publications believed to be of different authorship – and, the third separate the three playwright's known works with the contested ones removed. SPSS is used to conduct testing.

The Hierarchical Cluster Analysis technique uses Ward's Method with Squared Euclidean distance measurement, and nearest neighbor using both Squared Euclidean distance and Cosine options. The data is forced into three clusters for each playwright, Shakespeare, Marlowe, and Cary to observer where the chunks cluster.

Exploratory Factor Analysis (EFA), known as iterative PCA is conducted on the 57 chunks to optimize the RPAS algorithm. EFA aims to reduce the variables in the data into a smaller set of factors that explain the pattern of the relationships between the variables (Burns and Burns, 2008:443). By setting the threshold to 0.30 the most non-significant RPAS variable is removed and the data retested in an iterative process until the maximum variation in the data is explained by the eigenvalues. Once this is achieved, we use the identified components, also known as factors, for each of the significant variables that make up the components (factors) to plot the 57 chunks and observe how the known and contested works visually cluster. We test the data initially by using the Kaiser-Meyer-Olkin (KMO) to measure of sampling adequacy to ensure the value is greater than 0.5 and acceptable. We also ensure that Bartlett's Test of Sphericity has a significance value less than 0.05 indicating there are some relationships between the variables so that PCA can extract them. We apply Kaiser's criterion rule by producing as scree plot which highlights all of the eigenvalues and suggests retaining only factors that are above the eigenvalue of 1.

Stepwise Linear Discriminant Analysis (LDA) as an alternate classification technique to PCA is conducted (Balakrishnama and Ganapathiraju, 1998; Ye et al., 2004). We remove the contested works from the data and categorize all of the individual known authors' chunks, numbering them 1–3 and train the model. Using the resultant coefficients from the three Canonical Discriminant Functions, we plot the functions and compare the clusters.

Finally, we test the effectiveness of the algorithm. Rather than use k-fold cross-validation to test the accuracy of the model (Rodriguez et al., 2010), we draw on the full and partial synthetic data approach by Little (1993) and Rubin (1993). We elect to use the partial approach because we are not concerned with data disclosure (Drechsler et al., 2008). Five Shakespeare works are chosen at random and divided into 62 2000-word chunks. Five partially synthetic samples are constructed using 12 randomly selected chunks. Using the LDA resultant coefficients from the previous test, these new 24,000-word synthetic works are overlayed against the uncontested works to see how close they cluster to Shakespeare, Marlowe, and Cary.

## Results

Within this section, we discuss the correlation analysis results, the differences in the word accumulation curves, the hierarchical clustering, and PCA. We conclude with the stepwise LDA predictive model that is verified using a partial synthetic approach.

### Correlation analysis

The independence of the variables was tested using the Pearson correlation coefficient, “r” (see Table 2) and determined for the four high-level elements, Richness (R), Personal pronouns (P), Referential Activity Power (A), and Sensory Adjectives (S). The results were significant at the 0.01 level, with most of the relationships between the variables being deemed as weak or random (13–33%). Richness appeared to have a moderate to high correlation with Referential Activity Power, and the relationship bordered an inverse moderate to substantial level as it predicted around 69% of Referential Activity Power. In all cases, the relationship between Referential Activity Power and all other variables had an inverse relationship. Overall, the elements were independent of each other.

**Table 2:** Pearson correlation coefficient, R, results of RPAS, the five Sensory elements (VAHOG), and the four Referential Activity Power elements.

**Correlations**						
		**R**	**P**	**A**	**S**	
Richness (R)	Pearson Correlation	1	0.399^**^	−0.833^**^	0.456^**^	
	Sig. (2-tailed)		0.002	0	0	
	N	57	57	57	57	
Personal_Pronouns (P)	Pearson Correlation	0.399^**^	1	−0.451^**^	0.366^**^	
	Sig. (2-tailed)	0.002		0	0.005	
	N	57	57	57	57	
RA Power (A)	Pearson Correlation	−0.833^**^	−0.451^**^	1	−0.575^**^	
	Sig. (2-tailed)	0	0		0	
	N	57	57	57	57	
Sensory (S)	Pearson Correlation	0.456^**^	0.366^**^	−0.575^**^	1	
	Sig. (2-tailed)	0	0.005	0		
	N	57	57	57	57	
		**V**	**A**	**H**	**O**	**G**
Sensory-Visual (V)	Pearson Correlation	1	0.284^*^	0.715^**^	0.784^**^	0.571^**^
	Sig. (2-tailed)		0.032	0	0	0
	N	57	57	57	57	57
Sensory-Auditory (A)	Pearson Correlation	0.284^*^	1	−0.038	0.167	−0.119
	Sig. (2-tailed)	0.032		0.777	0.215	0.378
	N	57	57	57	57	57
Sensory-Haptic (H)	Pearson Correlation	0.715^**^	−0.038	1	0.632^**^	0.772^**^
	Sig. (2-tailed)	0	0.777		0	0
	N	57	57	57	57	57
Sensory-Olfactory (O)	Pearson Correlation	0.784^**^	0.167	0.632^**^	1	0.628^**^
	Sig. (2-tailed)	0	0.215	0		0
	N	57	57	57	57	57
Sensory-Gustatory (G)	Pearson Correlation	0.571^**^	−0.119	−0.119	0.628^**^	1
	Sig. (2-tailed)	0	0.378	0.378	0	
	N	57	57	57	57	57
		**A**	**C**	**P**	**PRON**	
RA Power-Article (A)	Pearson Correlation	1	0.800^**^	0.899^**^	0.686^**^	
	Sig. (2-tailed)		0	0	0	
	N	57	57	57	57	
RA Power-Conjunctive (C)	Pearson Correlation	0.800^**^	1	0.859^**^	0.563^**^	
	Sig. (2-tailed)	0		0	0	
	N	57	57	57	57	
RA Power-Preposition (P)	Pearson Correlation	0.899^**^	0.859^**^	1	0.706^**^	
	Sig. (2-tailed)	0	0		0	
	N	57	57	57	57	
RA Power-Pronoun (PRON)	Pearson Correlation	0.686^**^	0.563^**^	0.706^**^	1	
	Sig. (2-tailed)	0	0	0		
	N	57	57	57	57	

* *Correlation is significant at the 0.05 level (2-tailed)*.

** *Correlation is significant at the 0.01 level (2-tailed)*.

Pearson's correlation testing was conducted on the sensory adjectives that made up the Sensory element: Auditory, Gustatory, Haptic, Olfactory, and Visual. The results were significant at the 0.05–0.01 level. Of the five senses, Auditory was the weakest with either no correlation or a small random predictor relationship of 8%. Visual had the most number of correlations, but it had a weak to moderate relationship to all of the other sensory variables (varies between 8–61%). Gustatory, Olfactory, and Haptic had the same correlations and did not have a significant relationship to Auditory. They also had a weak to moderate relationship to all other sensory variables (varies between 33–60%). Again, the elements were independent of each other.

Pearson's correlation coefficient testing was used to determine the independence of the four linguistic variables known as particles that create the Referential Activity Power variables: Articles, Conjunctives, Prepositions, and Pronouns. The results were significant at the 0.01 level. The analysis showed that Prepositions are substantial as shown by its relationship with Articles (80.8%) and Conjunctives (73.8%) but not with pronouns (50%), and the relationship was only moderate. The correlation between Pronouns with Articles (47%) and Conjunctives (32%) highlight they were less correlated with a weak to moderate relationship. In this case, it would seem overall that the elements were less independent of each other.

### Word accumulation curves

**Figure 1:**
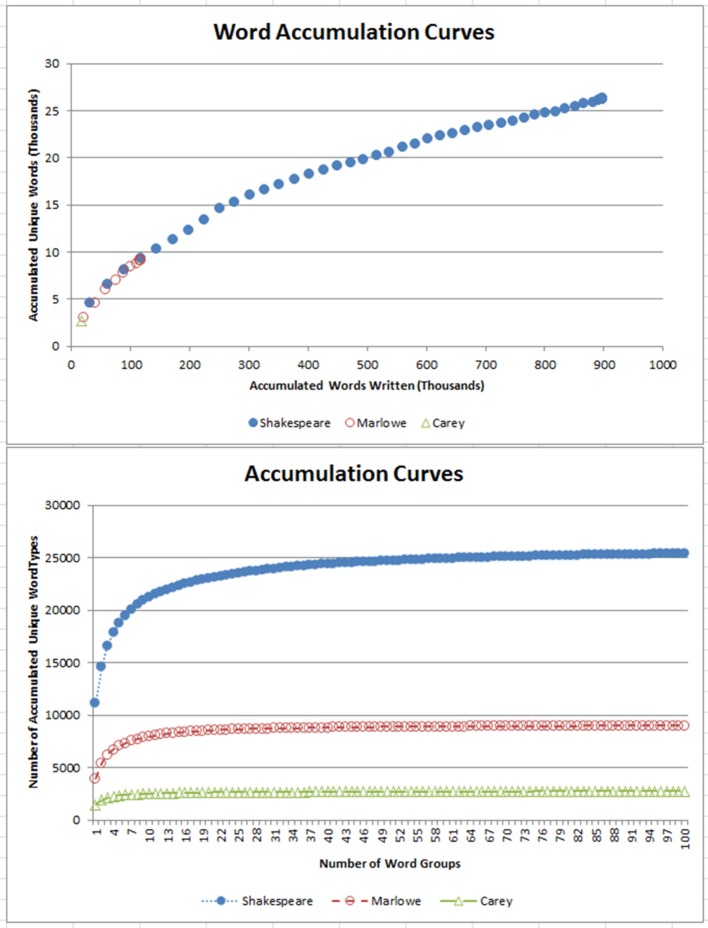
Word Accumulation Curves for Shakespeare, Marlowe, and Cary by word groups and accumulated words. In the **(lower)**, the different number of words each playwright used is shown and is different, but in the **(upper)**, the similarities between Marlowe and Shakespeare's word usage is highlighted.

There is a significant difference in the sample sizes of Shakespeare, Marlowe, and Cary. Therefore, as an alternate test for the Richness calculations, Word Accumulation Curves were plotted for Shakespeare's 897,308-word, Marlowe's 116,446-word, and Cary's 17, 376-word corpus to examine if their use of vocabulary was similar. As can be seen (lower panel Figure 1) Shakespeare's unique word list reached an asymptote at about the 50th largest word group, which is a total of 24,726 unique words. Marlowe's unique word list reached an asymptote at about the 21st largest word group, a total of 8,565 unique words, and Cary's unique word list reached an asymptote at about the 15th largest word group with a total of 2,599 unique words. When we compared the point where both word group curves asymptote, we could see Marlowe used about 34.6% fewer unique words than Shakespeare. Cary used about 89.5% fewer words than Shakespeare. However, there is a significant difference between the number of works each produced, and comparisons of the word accumulation plots tell a different story (upper panel Figure 1). It highlighted that Marlowe and Shakespeare have similar word growth that might take into account the influence of vocabulary size. We cannot make a comparison with Cary with a single work. There is an age difference between Shakespeare and Marlowe which could account for these differences. People's vocabulary is known to peak late in adulthood before it declines (currently peaking around 65 years. See Hartshorne and Germine, 2015), but this could highlight that age differences contribute to and help differentiate people from their Richness scores.

Of all the works of Shakespeare over 10,000 words, the unique words contributed about 13–23% (2400–4600 words). About 45% of these words are of the small group of 450 function words that account for less than 0.1 percent of the English vocabulary but make up more than half of the words commonly used (Pennebaker, 2011). Of all of the works of Marlowe over 10,000 words, the unique words contributed about 14–20% (2700–3200 words), and 42% are function words. In both cases, the chunks are well below a size that would approach the asymptote, and we deem that this phenomenon occurs outside of our enforced limit of a 30,000-word sample.

### Hierarchical clustering (HC)

To determine if there are differences in the writing styles of the three playwrights, the data was forced into three clusters using Hierarchical Cluster Analysis, (using Ward's Method with Squared Euclidean distance measure, and nearest neighbor using both Squared Euclidean distance and Cosine measure). It was expected that by forcing three clusters, one for each playwright (Shakespeare, Marlowe, and Cary), they would appear in separate clusters. However, the data variations in the contested and non-contested authored works were too distant in Euclidean space, and one of the clusters that formed had all three playwrights in them (see Table S1 External Data). Another test would need to be performed on a smaller set of the data without the contested, non-authored works, therefore as an alternative, PCA was conducted.

### Principal component analysis (PCA)

Iterative PCA was conducted to optimize the algorithm by the maximum variance explained by eigenvalues was conducted. Initially, PCA was conducted on the four high-level variables, Richness, Personal Pronouns, Referential Activity Power, and Sensory Adjectives. Only one factor was extracted and accounted for 64.3% of the variance. All the remaining three factors accounted for (35.78%) and were not significant.

PCA was extended, and the Referential Activity Power element was substituted with its four variables. Articles, Conjunctives, Prepositions, and Pronouns were tested to determine if the total variance would increase over the initial 63.4% obtained from the single factor. However, only one factor was extracted, and it accounted for 65.6% of the variance. All the remaining six factors accounted for 34.4% and were not significant. Overall, the total variance explained by the single factor increased by 1.3% over the initial test.

PCA was again extended, and the Sensory element was substituted with its five variables. Now, with the Visual, Auditory, Haptic, Olfactory, and Gustatory (VAHOG) variables, many correlations were more than 0.30, and both the KMO and Bartlett's tests produced criteria that support the application of PCA (0.722, *p* < 0.001). Communalities varied from 0.842 to 0.354. By applying Kaiser's Rule and scree test, two factors were deemed important. Following rotation, factor one was loaded on five items that reflect four of the five sensory elements variables and RA Power accounted for 49.56% of the variance. Factor two is loaded on the Richness, personal pronouns, RA Power, and two of the Sensory adjectives (Auditory and Visual) and accounted for 22.32% of the variance. Overall, the total variance explained by the two factors was 71.88%. These results show an increase of 7.6% over the initial test and 6.3% better than the second test that expanded the Referential Activity Power elements. Unweighted least squares Factor Analysis results highlighted Pearson's r correlations and indicated the inverse nature of Referential Activity Power along with the isolated Auditory variable. The Correlation Matrix, KMO, and Bartlett's Test, Communalities, Total Variance Explained, Scree Plot, and Component Matrix results are found in the External Data.

**Figure 2:**
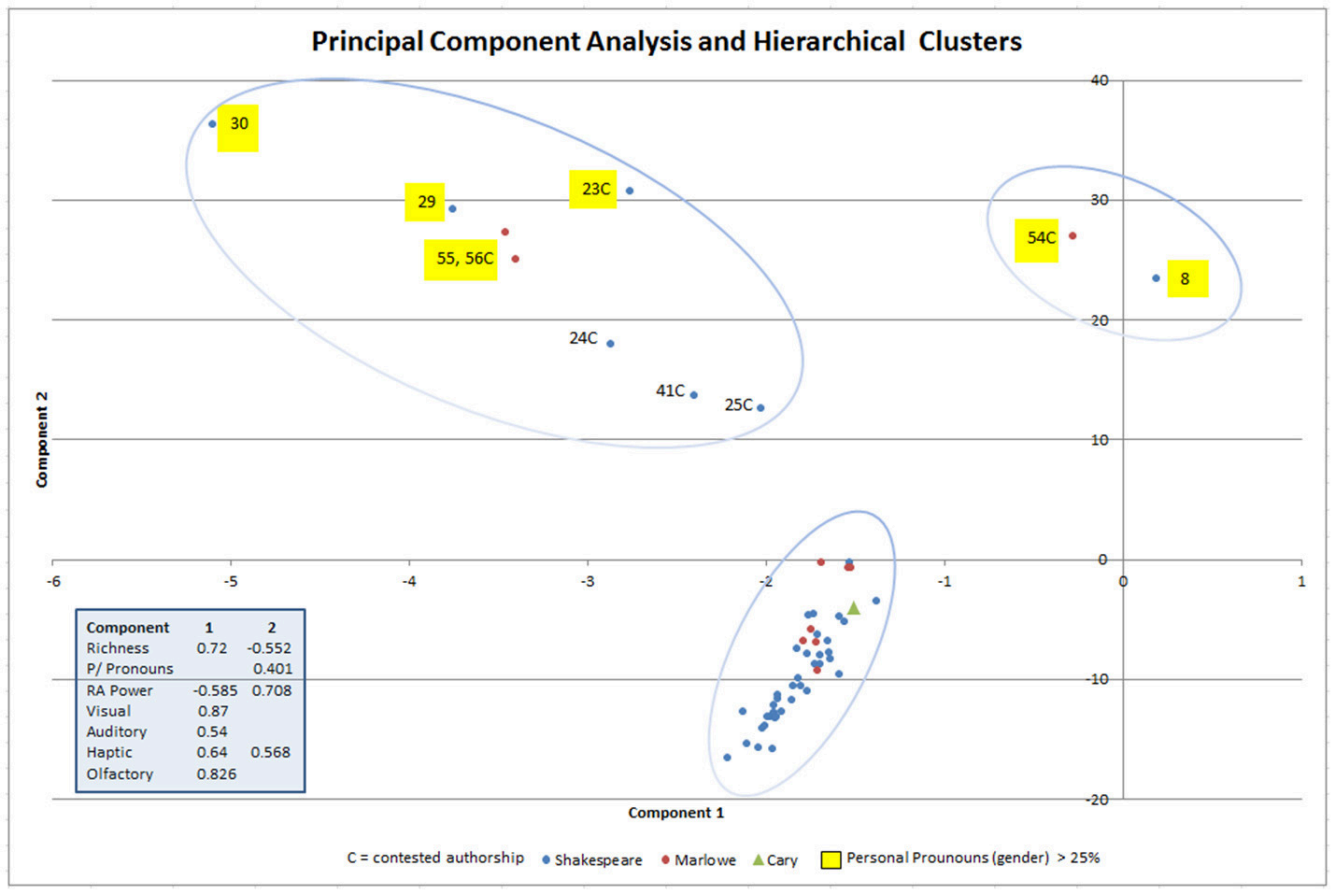
Results of the two clusters from the Principal Component Analysis overlayed with the Hierarchical Cluster Analysis results and showing the three clusters that form to separate the known works of the three playwrights from the works that are of contested authorship (or in the case of 8, 29, and 30 are stylistically different). The Personal Pronoun (gender) scores where they are > 0.25 are also shown to emphazise differences. The table highlights the contribution of the two components that the RPAS-VAHOG variables made.

The results of the Hierarchical Clustering and the PCA can be overlaid to reinforce the consistency of the results (Figure 2) and show the separation of the contested works from the main body of works. This was identified through the two leading factors of the PCA grouped by the Hierarchical Clustering results (blue ellipses). These methods are robust enough to correlate precisely. The cluster at the bottom contains most of the chunks for all three authors. The second largest cluster on the top left contains works of uncertain or mixed authorship, such as Shakespeare's *The Passionate Pilgrim* (chunks 23-25, and 41), and Marlowe's two-authored *The Passionate Shepherd to his Love* (chunks 55-56). The exception was Shakespeare's *The Phoenix and the Turtle* (chunks 29-30). While the differences in *The Phoenix and the Turtle* have been put down to Shakespeare's genius (Bednarz, 2012) and there is still some uncertainty over authorship (Richards, 1958), it is an accepted Shakespearian work. The cluster on the top right showed one work each of Shakespeare and Marlowe's that are stylistically quite different from their other works. Chunk 54 for example, *Hero and Leander*, was completed by George Chapman after Marlowe's death (Williams, 2005). *Venus and Adonis* was suggested to be written during Shakespeare's hard times during the plague (Stritmatter, 2004), and it is said to lack a sense of form and seen as dull (Putney, 1941). The results were reinforced by the personal pronoun analysis. Here we highlighted that most works are low in this category, and seven chunks had scores over 25% (Figure 2 yellow boxes highlight chunks 8, 23, 29–30, and 54–56). Two of these are high scores (>80%) and appeared in the top right cluster. When comparing Richness against Referential Activity Power, four very noticeable spikes occur (chunks 24, 29-30, 41, and 55-56), and these were also the works that appear in the top left cluster. Two lesser spikes occurred in the top right cluster (8 and 54). This relationship between Richness and Referential Activity Power is unusual and discussed further below. To further reinforce these consistent results, analysis of Richness against Sensory identified a large cluster of Shakespeare and Marlowe's works, but this time with a diffuse set of outliers. Most of these outliers were the same as those in the top clusters in Figure 2. For PCA results refer to Tables 1–5 and Figure 1 in External Dataset.

### Stepwise linear discriminant analysis (LDA)

**Figure 3:**
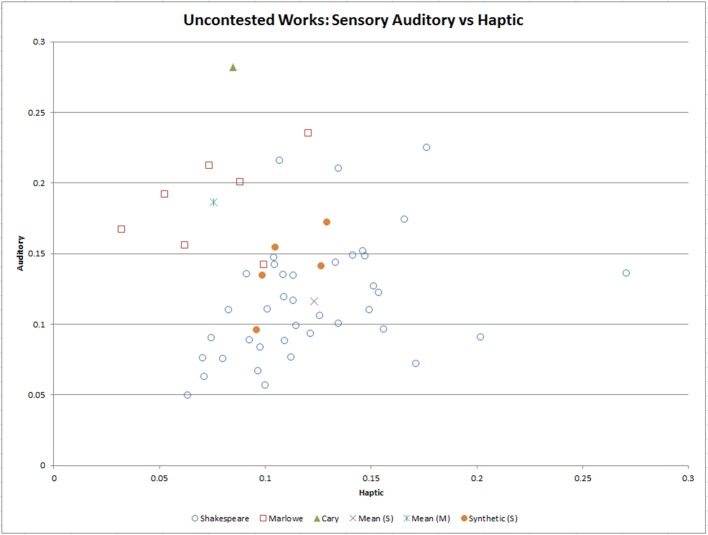
Results of the Linear Discriminant Analysis of the uncontested works of the playwrights showing the most significant element from each canonical function (Auditory and Haptic Sensory elements). The mean of the works of each playwright is also shown. After constructing five partially synthetic Shakespeare works and overlaying them against the original data, they are closest to Shakespeare.

To look at the data in more detail, the contested works were removed from the data, and stepwise LDA conducted. LDA is better at data classification than PCA, and it is less susceptible to shape and location changes when transformed to different spaces than PCA (Ye et al., 2004). The results of LDA on the eleven elements showed that three variables contributed the most to the classification of the data: Auditory, Haptic, and Richness. Two canonical discriminant functions were extracted, and both were statistically significant (*p* < 0.001, and *p* = 0.002), as was shown in the Wilks' Lambda results (refer to External Data). The Canonical Discriminant Functions plot of each playwright also highlighted clear separation in their centroids. Using this information, we reviewed the two sensory elements, Haptic against Auditory, and Richness against Auditory to discriminate the works of each playwright. Figure 3 shows the work chunks clustered against the Auditory and Haptic sensory elements. From the group centroids, there was a clear separation of the authors. Overall, Shakespeare's chunks had a style that was higher than Marlowe in the Haptic element (0.13 vs. 0.08), and lower in Auditory (0.12 vs. 0.19) and Richness (15.5 vs. 18) with the auditory signature being a very strong separator.

To further test the effectiveness of the algorithm, five Shakespeare works were chosen at random (chunks 6, 14, 19, 33, and 37) and divided into 62 chunks (each of 2,000 words). Five synthetic samples were each constructed from 12 randomly selected chunks. These new 24,000-word synthetic works were overlayed against the uncontested works. As can be seen in the Haptic and Auditory plot (Figure 3), they visually aligned closer in style to Shakespeare, and their group centroid was closer in three-dimensional Euclidean space to Shakespeare than Marlowe (a distance of 31.7 vs. 34.2). For LDA results refer to Tables 6–9 and Figure 2 in External Dataset.

**Figure 4:**
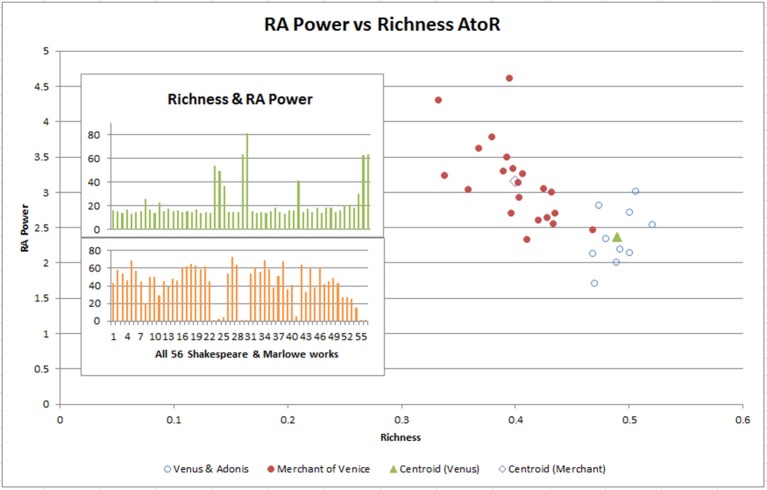
The Venus and Adonis play (8) which seems to be stylistically different and has an unusual Richness to Referential Activity Power relationship (see inset) is divided into 2,000 word chunks as is the Merchant of Venice (16). The centroids of each play maintain the low RA Power/high Richness anomaly, highlighting the results in the inset is not an artifact of the size of the play.

As mentioned earlier, the relationship between Richness and Referential Activity Power is unusual. Referential Activity Power (A) is formed using function words (highly “concrete” and “image-laden” pronouns, articles, conjunctives, and prepositions) from the Medical Research Council (MRC) Psycholinguistic database (Coltheart, 1981). It is used to identify a person's level of depression by using Referential Activity words (Bucci and Kabasakalian-McKay, 2004). We superimposed this against *Richness* (R), a valuable stylistic contributor for authorship identification from Menhinick's Index used to measure species diversity (Menhinick, 1964). This RA Power to Richness (AtoR) mapping (Figure 4 inset) highlighted several works with stylistic features likely written during difficult periods of the playwright's lives, perhaps brought about from the Bubonic Plague closing theaters, and against a backdrop of a poor economic environment and violent conditions in London during the late 1590s. The two insets highlighted some Richness spikes (upper diagram) with low Referential Activity Power values (chunks 8, 23, 24, 25, 41, 55, 56). These higher Richness chunks were less concrete, more abstract and surreal, and they had less imagery and emotion across the sensory aspects, which highlighted a different style to the other works.

Of these, the only non-contested Shakespeare work, *Venus and Adonis* (chunk 8), was suggested to be written during Shakespeare's hard times during the plague (Stritmatter, 2004) and be dull and lack a sense of form (Putney, 1941). To remove any chunking bias, we resampled Shakespeare's *Venus and Adonis* and *Merchant of Venice* into 2000 word–sized chunks and plotted AtoR (Figure 4). We would have expected a lower RA Power (Bucci and Maskit, 2004) in a depressed state, which is what we observed in the centroid differences between the two works. We see Richness as a very strong separator. However, we would also have expected to see more lexical repetition through a lower Richness score (Garrard et al., 2005). It is possible that the work was an early collaboration with another author, which was why it appeared near Marlowe's collaboration with George Chapman (refer to top right cluster in Figure 2). It is also possible that the higher Richness was due to Shakespeare's large vocabulary.

## Discussion

Using modern techniques on 400-year old data has some limitations. After William the Conqueror invaded England, Anglo-Norman (French) became the administrative language of Kings and nobility in England for more than 300 years. However, Anglo-Saxon (Old) English use remained in 95% of peasants and the lower class and resurged due to the 100 Year War against France, and the earlier Bubonic Plague in the mid-fourteenth century. Shakespeare's Early Modern England emerged, borrowing over 10,000 Norman words, removing noun genders, simplifying adjective inflections, and The Great Vowel Shift commenced (Mastin, 2011), and pronunciation changed during 1350 to 1700. It marked the point at which language became more standardized and akin to today.

To further put the results into perspective, Early Modern English began around the sixteenth century when vocabulary expanded at its greatest rate, and it is much closer to today's language than that of Old or Middle English (Horobin, 2010). By this time pronouns, *they, their, them* had become firmly established in the standard language, such as most personal pronouns that have maintained number, case, and gender throughout the history of English. The word *its* only came into print in 1598, and *his* was a neuter possessive where today we would use *its* (Nevalainen, 2006). While we elected not to replace *its* with *his* words because while *its* does not appear in any copy of Shakespeare's works published during his lifetime, some instances do appear in his posthumous published plays. Replacing *its* with *his* would change the gender category of two poems, *A Lover's Complaint* (personal pronouns score moved from 0.03 to 0.96) and *The Rape of Lucrece* (personal pronouns score moved from 0.003 to 1). While *A Lover's Complaint* has been attributed to the poet John Davies of Hereford by Vickers (2007), Wilson (1988) says that *The Rape of Lucrece* occupies an uncertain position in Shakespeare's canon, as an early, apprentice, experimental piece. Our analysis before using the word *his* instead of *its* suggests that outside of the higher gender score from personal pronoun use, *The Rape of Lucrece* is a Shakespeare written poem, while *A Lover's Complaint* was a contested work not written by Shakespeare. Distinct sets of indefinite and definite articles and demonstratives also existed by this time and support our algorithm's success to define the self from RA Power also, any many of the 117 function words taken from the MRC Psycholinguistic database were used during this period. While the meaning of some words has changed over time, many of the sensory adjectives from the list were not identified, but there were enough early and simpler Early Modern English words identified to be of value.

Empirical Zipf distributions and word accumulation curves have been used to highlight differences in word frequency distribution between Old English and Modern English of about 23%, whereas the differences between Early Modern English and Modern English is around 10% with the two modern language distributions being similar in terms of case, marking, and other inflectional paradigms like subjunctive ones, which have been replaced today by modal verbs (Bentz et al., 2014). Language does change over time, as does the meaning of some words, but by applying our approach across all of the Elizabethan works only and not drawing on any modern English works, any bias is consistent and does not change the clustering results.

Estimating Shakespeare' word use for authorship identification purposes might be effective (see the Taylor poem in Thisted and Efron, 1987). It is known that Shakespeare had an active vocabulary of over 21,000 different words, and while today's educated person's vocabulary is less than half that, Shakespeare has been credited with introducing more than two thousand words into today's everyday use (Bragg, 2003). Shakespeare's strength was his support from the King, to write and perform his plays in the emerging trade center, London for all to hear, the impact akin to today's newspapers and the internet. Brown and Gilman (1989) suggest that Shakespeare's dramatic text provide the best information on the colloquial speech of the period. He represented the conduct within court and society during a rich period of cultural reform and loaned from a library of lost voices (Bristol, 1996). Shakespeare's works are overrepresented in the first edition of the Oxford English Dictionary, contributing almost 33,000 quotations (Hoffmann, 2004), and he would have leaned on existing words in use during this important period of language reform. Notwithstanding this, it was estimated that Shakespeare knew an additional 35,000 words he did not use (Efron and Thisted, 1976). Word accumulation curves (Figure 1) highlighted, that during his life Shakespeare used around 21% more unique words than Marlowe. However, there was a significant difference between the number of works each produced and a comparison of word accumulation plots highlight they have similar word growth that might take into account the influence of vocabulary size varying with age differences (Hartshorne and Germine, 2015). Regression Analysis showed similar Richness characteristics for Shakespeare and Marlowe, and results of two-sample *T*-Tests (*p*-value 0.980) also suggested no significant difference between Shakespeare and Marlowe when Johnson Arcsine Transformations are applied to normalize the positively skewed data. Therefore, we suggest Richness (R) is a valuable stylistic contributor for authorship identification.

The correlation analysis of the four high-level RPAS variables highlighted that the RPAS variables are best used in this configuration, or as RPAS(VAHOG) without the five independent sensory elements aggregated into one Sensory Adjective (S) variable. This was also highlighted in the results of the LDA.

There were also some periods of “depression-like” episodes identified in the playwrights where RA Power dips predominantly (as shown by AtoR in Figure 4). These results are also reflected in the sensory-based adjectives, and might be useful in determining changes in the cognitive states of people, and has the potential to identify characteristics of self within cyberspace for law enforcement purposes.

## Conclusions

We find RPAS, the use of Richness (R), personal pronouns (P), RA Power (A), and sensory-based adjectives (S) is a different approach to the identification of self. It includes words that are strong in concreteness and imageability that reflect known psychological states in an individual's personality. The use of “sotto voce,” the authorial voice which projects the true identity of the authors has enabled us to separate Shakespeare's works. The broader implications of this research may provide signaling of depressive episodes that could have major social implications, such as averting suicide.

## Author contributions

RB and TB: Designed the study; DK: Processed the data and created the author signatures. All authors analyzed the results and contributed equally to the writing of the paper.

### Conflict of interest statement

The authors declare that the research was conducted in the absence of any commercial or financial relationships that could be construed as a potential conflict of interest.
