# Supplementary material for: Using Shakespeare's Sotto Voce to Determine True Identity From Text
Source: Front Psychol. 2018 Mar 15;9:289. doi: 10.3389/fpsyg.2018.00289 (PMC5862847; doi:10.3389/fpsyg.2018.00289)
Supplement: Supplementary file 1 [file Table1.DOCX]

Supplementary Material

Using Shakespeare’s Sotto Voce to Determine True Identity from Text

**David Kernot*, Terry Bossomaier, Roger Bradbury**

*** Correspondence:** Corresponding Author: u5604766@anu.edu.au

# Supplementary Data

**TableS1: Hierarchical Cluster Analysis Membership for 3 clusters**

| **Cluster Membership** | | | | | | | |
| --- | --- | --- | --- | --- | --- | --- | --- |
| **Case** | | **3 Clusters** | **Case** | | **3 Clusters** | **Case** | **3 Clusters** |
| 1:C1 | | 1 | 20:C7 | | 1 | 39:T9 | 1 |
| 2:H1 | | 1 | 21:C9 | | 1 | 40:C14 | 1 |
| 3:H2 | | 1 | 22:T3 | | 1 | 41:P8 | 3 |
| 4:H3 | | 1 | 23:P5 | | 3 | 42:C15 | 1 |
| 5:H4 | | 1 | 24:P4 | | 3 | 43:P9 | 1 |
| 6:C2 | | 1 | 25:P3 | | 3 | 44:C16 | 1 |
| 7:T1 | | 1 | 26:C8 | | 1 | 45:C17 | 1 |
| 8:P1 | | 2 | 27:T4 | | 1 | 46:H10 | 1 |
| 9:C4 | | 1 | 28:C10 | | 1 | 47:CM1 | 1 |
| 10:T2 | | 1 | 29:P6 | | 3 | 48:CM2 | 1 |
| 11:P2 | | 1 | 30:P7 | | 3 | 49:CM3 | 1 |
| 12:C3 | | 1 | 31:C11 | | 1 | 50:CM4 | 1 |
| 13:C5 | | 1 | 32:C12 | | 1 | 51:CM5 | 1 |
| 14:H5 | | 1 | 33:C13 | | 1 | 52:CM6 | 1 |
| 15:H6 | | 1 | 34:T5 | | 1 | 53:CM7 | 1 |
| 16:C6 | | 1 | 35:T6 | | 1 | 54:CM8 | 2 |
| 17:H7 | | 1 | 36:T7 | | 1 | 55:CM9 | 3 |
| 18:H8 | | 1 | 37:T10 | | 1 | 56:CM10 | 3 |
| 19:H9 | | 1 | 38:T8 | | 1 | 57:EC1 | 1 |
|  | | |  |  |  |  |  |
