# Supplementary material for: Using Shakespeare's Sotto Voce to Determine True Identity From Text
Source: Front Psychol. 2018 Mar 15;9:289. doi: 10.3389/fpsyg.2018.00289 (PMC5862847; doi:10.3389/fpsyg.2018.00289)
Supplement: Supplementary file 2 [file Table2.DOCX]

Supplementary Material

Using Shakespeare’s Sotto Voce to Determine True Identity from Text

**David Kernot*, Terry Bossomaier, Roger Bradbury**

*** Correspondence:** Corresponding Author: u5604766@anu.edu.au

# Supplementary Data

Table S2: Shakespeare and Marlowe's data

| Work | C1 | H1 | H2 | H3 | H4 |
| --- | --- | --- | --- | --- | --- |
| Richness | 16.25 | 15.3 | 14.04 | 17.05 | 13.14 |
| Gender | 0 | 0 | 0 | 0 | 0 |
| MF | F | F | F | F | F |
| RA Score | 42.7314 | 57.6725 | 53.8094 | 45.9779 | 68.7998 |
| Visual | 0.722373 | 0.588391 | 0.445697 | 0.672587 | 0.75609 |
| Auditory | 0.126896 | 0.135264 | 0.093529 | 0.135581 | 0.21018 |
| Haptic | 0.151254 | 0.108418 | 0.1213 | 0.090989 | 0.134542 |
| Olfactory | 0.103741 | 0.059216 | 0.124116 | 0.059422 | 0.062002 |
| Gustatory | 0.113318 | 0.09229 | 0.138024 | 0.073384 | 0.107353 |
| Work | C2 | T1 | P1 | C4 | T2 |
| Richness | 14.81 | 15.76 | 25.56 | 17.08 | 14.29 |
| Gender | 0 | 0 | 100 | 0 | 0 |
| MF | F | F | M | F | F |
| RA Score | 57.4333 | 45.5241 | 20.4723 | 49.9042 | 49.8822 |
| Visual | 0.541181 | 0.73169 | 1.723258 | 0.721879 | 0.902494 |
| Auditory | 0.122363 | 0.134754 | 0.234055 | 0.090929 | 0.225057 |
| Haptic | 0.153726 | 0.113236 | 0.466293 | 0.201918 | 0.176187 |
| Olfactory | 0.127216 | 0.171473 | 0.336242 | 0.331369 | 0.189831 |
| Gustatory | 0.147916 | 0.169366 | 0.338636 | 0.242046 | 0.146719 |
| Work | P2 | C3 | C5 | H5 | H6 |
| Richness | 22.76 | 15.14 | 17.55 | 15.6 | 16.41 |
| Gender | 0.32 | 0 | 0 | 0 | 0 |
| MF | F | F | F | F | F |
| RA Score | 29.3536 | 45.2309 | 39.11 | 48.2981 | 46.3632 |
| Visual | 1.636045 | 0.398482 | 0.902377 | 0.777078 | 0.577187 |
| Auditory | 0.136004 | 0.110695 | 0.143564 | 0.151687 | 0.110339 |
| Haptic | 0.270676 | 0.100975 | 0.133363 | 0.14608 | 0.149137 |
| Olfactory | 0.201118 | 0.139795 | 0.28737 | 0.103742 | 0.075876 |
| Gustatory | 0.179946 | 0.147698 | 0.201419 | 0.152251 | 0.116437 |
| Work | C6 | H7 | H8 | H9 | C7 |
| Richness | 14.79 | 15.1 | 15.03 | 16.82 | 13.66 |
| Gender | 0 | 0 | 0 | 0 | 0 |
| MF | F | F | F | F | F |
| RA Score | 60.7166 | 62.4006 | 65.2883 | 63.0035 | 59.2003 |
| Visual | 0.511247 | 0.58832 | 0.684248 | 0.647231 | 0.323587 |
| Auditory | 0.08393 | 0.100553 | 0.148306 | 0.116549 | 0.090223 |
| Haptic | 0.097446 | 0.134597 | 0.147304 | 0.113065 | 0.074477 |
| Olfactory | 0.106484 | 0.10739 | 0.094581 | 0.053971 | 0.104642 |
| Gustatory | 0.108136 | 0.119524 | 0.102143 | 0.078758 | 0.09144 |
| Work | C9 | T3 | P5 | P4 | P3 |
| Richness | 14.54 | 13.91 | 54.05 | 49.23 | 37.1 |
| Gender | 0 | 0 | 53.74 | 0.23 | 0.29 |
| MF | F | F | M | F | F |
| RA Score | 61.7189 | 44.9894 | 0.73211 | 1.87868 | 4.28841 |
| Visual | 0.51063 | 0.435933 | 0.103728 | 1.312949 | 1.17865 |
| Auditory | 0.05654 | 0.088547 | 0.170205 | 0.284725 | 0.152885 |
| Haptic | 0.099978 | 0.109114 | 0.117218 | 0.192323 | 0.389512 |
| Olfactory | 0.087456 | 0.035437 | 0 | 0.40099 | 0.307529 |
| Gustatory | 0.130911 | 0.080424 | 0.110089 | 0.239336 | 0.347414 |
| Work | C8 | T4 | C10 | P6 | P7 |
| Richness | 14.99 | 14.79 | 14.67 | 63.7 | 81.33 |
| Gender | 0 | 0 | 0 | 28.29 | 28.29 |
| MF | F | F | F | F | F |
| RA Score | 53.5463 | 73.3452 | 64.1068 | 0.95537 | 0.17258 |
| Visual | 0.480171 | 0.506606 | 0.661711 | 0.763579 | 0.511727 |
| Auditory | 0.075384 | 0.147291 | 0.07589 | 0.233244 | 0.839677 |
| Haptic | 0.080101 | 0.104015 | 0.070321 | 0 | 0 |
| Olfactory | 0.103438 | 0.084115 | 0.068191 | 0 | 0 |
| Gustatory | 0.098992 | 0.096778 | 0.076608 | 0 | 0 |
| Work | C11 | C12 | C13 | T5 | T6 |
| Richness | 15.67 | 14.31 | 14.59 | 13.62 | 15.13 |
| Gender | 0 | 0 | 0 | 0 | 0 |
| MF | F | F | F | F | F |
| RA Score | 54.4096 | 61.2111 | 55.6308 | 68.9821 | 59.1706 |
| Visual | 0.605774 | 0.294831 | 0.304151 | 0.463054 | 0.530729 |
| Auditory | 0.071912 | 0.066786 | 0.062685 | 0.099035 | 0.10621 |
| Haptic | 0.171197 | 0.096414 | 0.071046 | 0.114678 | 0.125608 |
| Olfactory | 0.169386 | 0.074955 | 0.04193 | 0.14299 | 0.050604 |
| Gustatory | 0.200577 | 0.094433 | 0.04786 | 0.084359 | 0.111693 |
| Work | T7 | T10 | T8 | T9 | C14 |
| Richness | 18.24 | 14.8227 | 13.59 | 16.51 | 16.47 |
| Gender | 0 | 0 | 0 | 0 | 0 |
| MF | F | F | F | F | F |
| RA Score | 37.5829 | 51.44967 | 67.7833 | 36.3134 | 41.235 |
| Visual | 0.67259 | 0.546129 | 0.271527 | 0.712465 | 0.604835 |
| Auditory | 0.076665 | 0.21603 | 0.049908 | 0.119291 | 0.142063 |
| Haptic | 0.112186 | 0.106733 | 0.06322 | 0.108834 | 0.104375 |
| Olfactory | 0.062566 | 0.04106 | 0.048431 | 0.048913 | 0.069686 |
| Gustatory | 0.055278 | 0.074547 | 0.051461 | 0.067366 | 0.06346 |
| Work | P8 | C15 | P9 | C16 | C17 |
| Richness | 41.2 | 14.43 | 17.37 | 14.6 | 18.33 |
| Gender | 3.05 | 0 | 0 | 0 | 0 |
| MF | F | F | F | F | F |
| RA Score | 5.65147 | 63.4876 | 33.1499 | 60.5912 | 38.1909 |
| Visual | 1.146025 | 0.498959 | 1.037895 | 0.500051 | 0.43996 |
| Auditory | 0.109813 | 0.148758 | 0.17438 | 0.089062 | 0.096589 |
| Haptic | 0.32484 | 0.141516 | 0.165947 | 0.092333 | 0.155833 |
| Olfactory | 0.201539 | 0.069874 | 0.297425 | 0.075552 | 0.124506 |
| Gustatory | 0.284403 | 0.104483 | 0.24522 | 0.100852 | 0.11218 |
| Work | H10 |  |  |  |  |
| Richness | 13.96 |  |  |  |  |
| Gender | 0 |  |  |  |  |
| MF | F |  |  |  |  |
| RA Score | 61.0156 |  |  |  |  |
| Visual | 0.504383 |  |  |  |  |
| Auditory | 0.110081 |  |  |  |  |
| Haptic | 0.082795 |  |  |  |  |
| Olfactory | 0.045396 |  |  |  |  |
| Gustatory | 0.081841 |  |  |  |  |
| Christopher | Marlowe | Data |  |  |  |
| Works | 1 | 2 | 3 | 4 | 5 |
| Richness | 18.28552 | 18.09594 | 14.49615 | 16.02585 | 20.66846 |
| Gender | 0 | 0 | 0 | 0 | 0.004535 |
| MF | F | F | F | F | F |
| RA Score | 42.39866 | 45.18381 | 48.83392 | 42.96724 | 26.66785 |
| Visual | 1.000979 | 0.901912 | 0.482424 | 0.669131 | 0.64329 |
| Auditory | 0.234993 | 0.142154 | 0.200488 | 0.192025 | 0.167284 |
| Haptic | 0.120406 | 0.099446 | 0.088144 | 0.052525 | 0.031992 |
| Olfactory | 0.127664 | 0.151827 | 0.178488 | 0.065615 | 0.164927 |
| Gustatory | 0.090736 | 0.118565 | 0.146348 | 0.077117 | 0.130662 |
| Works | 6 | 7 | 8 | 9 | 10 |
| Richness | 19.62522 | 18.68243 | 30.51459 | 63.05732 | 63.31361 |
| Gender | 0.003707 | 0 | 0.833598 | 0.263845 | 0.276473 |
| MF | F | F | M | F | F |
| RA Score | 26.9586 | 25.45618 | 14.75284 | 0.799477 | 0.534205 |
| Visual | 0.937972 | 0.618672 | 1.407123 | 1.987261 | 0.982249 |
| Auditory | 0.212231 | 0.155906 | 0.162535 | 0.343949 | 0 |
| Haptic | 0.073649 | 0.061938 | 0.168026 | 0.273885 | 0.254438 |
| Olfactory | 0.192185 | 0.168031 | 0.258864 | 0.726115 | 0.189349 |
| Gustatory | 0.154489 | 0.127412 | 0.147474 | 0.229299 | 0.579882 |
