# Supplementary material for: Using Shakespeare's Sotto Voce to Determine True Identity From Text
Source: Front Psychol. 2018 Mar 15;9:289. doi: 10.3389/fpsyg.2018.00289 (PMC5862847; doi:10.3389/fpsyg.2018.00289)
Supplement: Supplementary file 3 [file Table3.DOCX]

Supplementary Material

Using Shakespeare’s Sotto Voce to Determine True Identity from Text

**David Kernot*, Terry Bossomaier, Roger Bradbury**

*** Correspondence:** Corresponding Author: u5604766@anu.edu.au

# Supplementary Data

Table S3: Referential Activity Power data

| WORD | TYPE | RA |  | WORD | TYPE | RA |
| --- | --- | --- | --- | --- | --- | --- |
| A | A | 0.29857 |  | ONCE | C | 0.455 |
| ABOUT | P | 0.32286 |  | ONE | PNOUN | 0.57929 |
| AFTER | P | 0.32786 |  | ONLY | P | 0.33786 |
| ALL | PNOUN | 0.42786 |  | OR | P | 0.33714 |
| ALTHOUGH | C | 0.30429 |  | OTHER | PNOUN | 0.38571 |
| AN | A | 0.29429 |  | OUR | PNOUN | 0.4 |
| AND | C | 0.31857 |  | OUT | P | 0.41357 |
| ANY | PNOUN | 0.30571 |  | OVER | P | 0.43857 |
| ANYBODY | PNOUN | 0.48 |  | OWN | PNOUN | 0.46714 |
| AS | PNOUN | 0.27286 |  | PAST | P | 0.52786 |
| AT | PNOUN | 0.29929 |  | POST | P | 0.75071 |
| BAR | P | 0.82929 |  | ROUND | P | 0.71214 |
| BECAUSE | C | 0.31429 |  | SAME | PNOUN | 0.41 |
| BEFORE | P | 0.36857 |  | SAVE | P | 0.485 |
| BEST | PNOUN | 0.43286 |  | SELF | PNOUN | 0.66071 |
| BOTH | PNOUN | 0.44286 |  | SHE | PNOUN | 0.62857 |
| BUT | PNOUN | 0.30929 |  | SIN | P | 0.51 |
| BY | P | 0.31214 |  | SINCE | P | 0.365 |
| CAUSE | C | 0.40643 |  | SO | C | 0.30143 |
| CROSS | P | 0.725 |  | SOME | PNOUN | 0.42 |
| DOWN | P | 0.57 |  | SOUTH | P | 0.58786 |
| EACH | PNOUN | 0.42 |  | STILL | C | 0.49929 |
| ELSE | C | 0.30714 |  | SUCH | PNOUN | 0.33357 |
| EVERY | PNOUN | 0.37929 |  | THAN | C | 0.28643 |
| EXTRA | P | 0.42786 |  | THAT | PNOUN | 0.32643 |
| FAILING | P | 0.51143 |  | THE | A | 0.31857 |
| FEW | PNOUN | 0.45857 |  | THEIR | PNOUN | 0.36571 |
| FOR | P | 0.32929 |  | THEM | PNOUN | 0.50857 |
| FORE | P | 0.45714 |  | THEN | C | 0.28143 |
| FORTH | P | 0.47 |  | THESE | PNOUN | 0.37643 |
| FROM | P | 0.31143 |  | THEY | PNOUN | 0.44857 |
| GIN | P | 0.85286 |  | THIS | PNOUN | 0.37071 |
| HE | PNOUN | 0.60786 |  | THOSE | PNOUN | 0.35786 |
| HER | PNOUN | 0.63071 |  | THROUGH | P | 0.42429 |
| HIM | PNOUN | 0.56357 |  | THWART | P | 0.47429 |
| HIMSELF | PNOUN | 0.43 |  | TILL | C | 0.52071 |
| HIS | PNOUN | 0.46929 |  | TO | P | 0.29929 |
| I | PNOUN | 0.67714 |  | UNDER | P | 0.53214 |
| IF | C | 0.30857 |  | UP | P | 0.565 |
| IN | P | 0.43714 |  | UPON | P | 0.46071 |
| INN | P | 0.83571 |  | US | PNOUN | 0.58929 |
| INTO | P | 0.40357 |  | VICE | P | 0.58 |
| IT | PNOUN | 0.38286 |  | WE | PNOUN | 0.47071 |
| ITS | PNOUN | 0.31286 |  | WHAT | PNOUN | 0.38714 |
| LESS | P | 0.39429 |  | WHEN | PNOUN | 0.32143 |
| LIKE | P | 0.45571 |  | WHERE | C | 0.365 |
| ME | PNOUN | 0.67214 |  | WHICH | PNOUN | 0.33714 |
| MINE | PNOUN | 0.69571 |  | WHILE | C | 0.36143 |
| MORE | P | 0.39143 |  | WHO | PNOUN | 0.35357 |
| MY | PNOUN | 0.39357 |  | WHOM | PNOUN | 0.38857 |
| NEAR | P | 0.53214 |  | WHY | C | 0.37 |
| NEXT | P | 0.46286 |  | WITH | P | 0.39643 |
| NONE | PNOUN | 0.50929 |  | WITHOUT | C | 0.40643 |
| NOR | C | 0.30929 |  | WITHOUT | P | 0.40643 |
| NOW | C | 0.41786 |  | YET | C | 0.34714 |
| OF | P | 0.28357 |  | YONDER | PNOUN | 0.49214 |
| OFF | P | 0.43357 |  | YOU | PNOUN | 0.55 |
| ON | P | 0.37857 |  | YOUR | PNOUN | 0.38929 |

List of function words and their RA Power values, where Type A = Article, C = Conjunctive, P = Preposition, and PNOUN = Pronoun.
