# Supplementary material for: Using Shakespeare's Sotto Voce to Determine True Identity From Text
Source: Front Psychol. 2018 Mar 15;9:289. doi: 10.3389/fpsyg.2018.00289 (PMC5862847; doi:10.3389/fpsyg.2018.00289)
Supplement: Supplementary file 4 [file Table4.DOCX]

Supplementary Material

Using Shakespeare’s Sotto Voce to Determine True Identity from Text

**David Kernot*, Terry Bossomaier, Roger Bradbury**

*** Correspondence:** Corresponding Author: u5604766@anu.edu.au

# Supplementary Data

Table S4: Sensory Adjectives data

| Word | Modality | Exclusivity |  | Word | Modality | Exclusivity |
| --- | --- | --- | --- | --- | --- | --- |
| abrasive | visual | 0.469512 |  | icy | visual | 0.35412 |
| abrasive | haptic | 0.433761 |  | immense | haptic | 0.640601 |
| absorbent | auditory | 0.401216 |  | immense | visual | 0.89823 |
| absorbent | visual | 0.479784 |  | insipid | gustatory | 0.379187 |
| aching | haptic | 0.647287 |  | insipid | auditory | 0.604839 |
| aching | haptic | 0.640741 |  | itchy | haptic | 0.62776 |
| acidic | gustatory | 0.494737 |  | itchy | haptic | 0.644599 |
| acidic | gustatory | 0.471178 |  | jagged | visual | 0.534435 |
| acrid | olfactory | 0.434641 |  | jagged | visual | 0.441176 |
| acrid | gustatory | 0.474619 |  | jammy | gustatory | 0.259601 |
| adhesive | haptic | 0.448666 |  | jammy | gustatory | 0.289428 |
| adhesive | haptic | 0.48072 |  | jingling | auditory | 0.480638 |
| alcoholic | olfactory | 0.437198 |  | jingling | auditory | 0.5 |
| alcoholic | gustatory | 0.422535 |  | juicy | gustatory | 0.301887 |
| amber | visual | 0.636364 |  | juicy | gustatory | 0.230769 |
| amber | visual | 0.749035 |  | khaki | visual | 0.644068 |
| aromatic | olfactory | 0.510582 |  | khaki | visual | 0.659722 |
| aromatic | olfactory | 0.62585 |  | large | visual | 0.484694 |
| astringent | gustatory | 0.462427 |  | large | visual | 0.441237 |
| astringent | olfactory | 0.402632 |  | laughing | visual | 0.515957 |
| azure | visual | 0.616935 |  | laughing | auditory | 0.522788 |
| azure | visual | 0.689498 |  | leathery | haptic | 0.329389 |
| babbling | auditory | 0.505102 |  | leathery | haptic | 0.377404 |
| babbling | auditory | 0.440514 |  | lemony | gustatory | 0.439331 |
| balmy | haptic | 0.356688 |  | lemony | olfactory | 0.655629 |
| balmy | haptic | 0.252492 |  | light | olfactory | 0.717131 |
| banging | visual | 0.385776 |  | light | haptic | 0.472362 |
| banging | auditory | 0.456422 |  | lilting | auditory | 0.639391 |
| barbecued | olfactory | 0.157601 |  | lilting | visual | 0.529727 |
| barbecued | olfactory | 0.180068 |  | lithe | visual | 0.727273 |
| barking | auditory | 0.536524 |  | lithe | visual | 0.416235 |
| barking | auditory | 0.530864 |  | long | visual | 0.665625 |
| beautiful | visual | 0.516971 |  | long | auditory | 0.668342 |
| beautiful | auditory | 0.699634 |  | loose | visual | 0.488491 |
| beeping | auditory | 0.615337 |  | loose | visual | 0.483333 |
| beeping | auditory | 0.623907 |  | loud | auditory | 0.570225 |
| beery | olfactory | 0.497076 |  | loud | visual | 0.497268 |
| beery | olfactory | 0.216354 |  | low | auditory | 0.7 |
| beige | visual | 0.785441 |  | low | visual | 0.569405 |
| beige | visual | 0.915493 |  | lukewarm | haptic | 0.518625 |
| big | visual | 0.748062 |  | lukewarm | gustatory | 0.348115 |
| big | visual | 0.392276 |  | lumpy | visual | 0.305147 |
| bitter | gustatory | 0.530026 |  | lumpy | haptic | 0.514234 |
| bitter | haptic | 0.483745 |  | lush | visual | 0.433628 |
| black | visual | 0.512195 |  | lush | gustatory | 0.265976 |
| black | visual | 0.877551 |  | meaty | visual | 0.453165 |
| black and white | visual | 0.879464 |  | meaty | gustatory | 0.299669 |
| black and white | visual | 0.73617 |  | mellow | auditory | 0.730612 |
| bland | gustatory | 0.499106 |  | mellow | gustatory | 0.44697 |
| bland | visual | 0.769811 |  | melted | visual | 0.427208 |
| blaring | auditory | 0.613181 |  | melted | visual | 0.230279 |
| blaring | auditory | 0.594901 |  | metallic | gustatory | 0.312989 |
| bleating | auditory | 0.516129 |  | metallic | visual | 0.534574 |
| bleating | auditory | 0.524476 |  | mild | gustatory | 0.470862 |
| bleeping | auditory | 0.593865 |  | mild | visual | 0.419825 |
| bleeping | auditory | 0.531507 |  | miniature | visual | 0.578947 |
| bloody | visual | 0.456057 |  | miniature | visual | 0.452214 |
| bloody | visual | 0.378486 |  | minty | gustatory | 0.422907 |
| blotchy | visual | 0.665595 |  | minty | olfactory | 0.502907 |
| blotchy | visual | 0.651389 |  | moaning | auditory | 0.586806 |
| blue | visual | 0.932692 |  | moaning | auditory | 0.491139 |
| blue | visual | 0.910638 |  | moist | gustatory | 0.314841 |
| blunt | haptic | 0.541916 |  | moist | haptic | 0.318264 |
| blunt | haptic | 0.444604 |  | motionless | haptic | 0.391421 |
| boiling | auditory | 0.308772 |  | motionless | visual | 0.528395 |
| boiling | visual | 0.185185 |  | mottled | visual | 0.418457 |
| booming | auditory | 0.4447 |  | mottled | visual | 0.394191 |
| booming | auditory | 0.412664 |  | mouldy | visual | 0.39798 |
| bouncy | haptic | 0.477064 |  | mouldy | visual | 0.38322 |
| bouncy | visual | 0.436428 |  | muddy | visual | 0.341991 |
| branching | visual | 0.57622 |  | muddy | visual | 0.356137 |
| branching | visual | 0.606918 |  | murky | visual | 0.443031 |
| braying | auditory | 0.412616 |  | murky | visual | 0.461728 |
| braying | auditory | 0.493151 |  | murmuring | auditory | 0.608187 |
| breakable | visual | 0.417582 |  | murmuring | auditory | 0.515464 |
| breakable | visual | 0.412346 |  | mushroomy | visual | 0.508621 |
| breezy | auditory | 0.242126 |  | mushroomy | gustatory | 0.393574 |
| breezy | haptic | 0.371901 |  | mushy | haptic | 0.226714 |
| bright | visual | 0.981481 |  | mushy | haptic | 0.351145 |
| bright | visual | 0.707719 |  | musty | olfactory | 0.469208 |
| brilliant | visual | 0.729167 |  | musty | olfactory | 0.291221 |
| brilliant | auditory | 0.840164 |  | narrow | visual | 0.660436 |
| briny | gustatory | 0.353391 |  | narrow | visual | 0.511393 |
| briny | visual | 0.221631 |  | noisy | auditory | 0.545685 |
| bristly | haptic | 0.38785 |  | noisy | auditory | 0.759717 |
| bristly | haptic | 0.47773 |  | nutty | gustatory | 0.219055 |
| brittle | haptic | 0.391192 |  | nutty | gustatory | 0.412037 |
| brittle | haptic | 0.452309 |  | odorous | olfactory | 0.523929 |
| broad | visual | 0.543354 |  | odorous | olfactory | 0.510753 |
| broad | visual | 0.723164 |  | oily | gustatory | 0.272866 |
| broken | visual | 0.328814 |  | oily | visual | 0.389706 |
| broken | visual | 0.415842 |  | oniony | visual | 0.452872 |
| bronze | visual | 0.601329 |  | oniony | gustatory | 0.367311 |
| bronze | visual | 0.558912 |  | open | visual | 0.407336 |
| brown | visual | 0.736641 |  | open | visual | 0.335185 |
| brown | visual | 0.793358 |  | orange | visual | 0.91866 |
| bubbling | visual | 0.237288 |  | orange | visual | 0.395299 |
| bubbling | auditory | 0.3 |  | oval | visual | 0.508816 |
| bulky | visual | 0.474074 |  | oval | visual | 0.622951 |
| bulky | visual | 0.484485 |  | painful | auditory | 0.570621 |
| bumpy | haptic | 0.402 |  | painful | haptic | 0.557229 |
| bumpy | haptic | 0.450602 |  | pale | visual | 0.837302 |
| burning | visual | 0.37747 |  | pale | visual | 0.962617 |
| burning | olfactory | 0.217929 |  | patterned | visual | 0.713311 |
| burnt | haptic | 0.320704 |  | patterned | visual | 0.662539 |
| burnt | gustatory | 0.228614 |  | peachy | gustatory | 0.476427 |
| bursting | auditory | 0.404124 |  | peachy | visual | 0.525 |
| bursting | visual | 0.377593 |  | peppery | gustatory | 0.437367 |
| buttery | haptic | 0.454343 |  | peppery | gustatory | 0.375472 |
| buttery | gustatory | 0.281365 |  | perfumed | olfactory | 0.432432 |
| buzzing | auditory | 0.528455 |  | perfumed | olfactory | 0.501597 |
| buzzing | auditory | 0.539823 |  | petite | visual | 0.620178 |
| caramelised | gustatory | 0.2443 |  | petite | visual | 0.626546 |
| caramelised | gustatory | 0.241796 |  | pink | visual | 0.669903 |
| charred | visual | 0.271868 |  | pink | visual | 0.951111 |
| charred | visual | 0.200918 |  | plain | visual | 0.617555 |
| cheesy | gustatory | 0.289515 |  | plain | gustatory | 0.414894 |
| cheesy | olfactory | 0.479254 |  | plastic | visual | 0.355967 |
| chequered | visual | 0.794574 |  | plastic | haptic | 0.281955 |
| chequered | visual | 0.745923 |  | polished | visual | 0.417051 |
| chewy | gustatory | 0.334677 |  | polished | auditory | 0.682482 |
| chewy | gustatory | 0.314629 |  | popping | auditory | 0.414942 |
| chilly | haptic | 0.46832 |  | popping | auditory | 0.412998 |
| chilly | haptic | 0.428571 |  | portly | visual | 0.329377 |
| chiming | auditory | 0.559229 |  | portly | visual | 0.59542 |
| chiming | auditory | 0.60479 |  | prickly | haptic | 0.48642 |
| chirping | auditory | 0.665517 |  | prickly | haptic | 0.563177 |
| chirping | auditory | 0.585366 |  | puffy | haptic | 0.474299 |
| chocolatey | gustatory | 0.345576 |  | puffy | visual | 0.542029 |
| chocolatey | visual | 0.507808 |  | pulsing | visual | 0.702929 |
| chubby | visual | 0.517073 |  | pulsing | haptic | 0.467192 |
| chubby | visual | 0.490566 |  | pungent | olfactory | 0.537572 |
| circular | visual | 0.513661 |  | pungent | olfactory | 0.459854 |
| circular | visual | 0.433486 |  | purple | visual | 0.773723 |
| citrusy | olfactory | 0.48125 |  | purple | visual | 0.915584 |
| citrusy | gustatory | 0.4375 |  | purring | auditory | 0.483029 |
| clammy | haptic | 0.548387 |  | purring | auditory | 0.485175 |
| clammy | visual | 0.288381 |  | quiet | auditory | 0.553672 |
| clamorous | auditory | 0.36253 |  | quiet | auditory | 0.514986 |
| clamorous | auditory | 0.444444 |  | radiant | visual | 0.677656 |
| clanging | auditory | 0.537468 |  | radiant | visual | 0.413408 |
| clanging | auditory | 0.484988 |  | rancid | gustatory | 0.371041 |
| clean | olfactory | 0.3186 |  | rancid | olfactory | 0.455338 |
| clean | visual | 0.460526 |  | raspy | auditory | 0.891667 |
| clear | visual | 0.677193 |  | raspy | haptic | 0.385027 |
| clear | visual | 0.752727 |  | rectangular | visual | 0.512887 |
| clicking | auditory | 0.455847 |  | rectangular | visual | 0.788491 |
| clicking | auditory | 0.486216 |  | red | visual | 0.845833 |
| cloudy | visual | 0.536634 |  | red | visual | 0.567568 |
| cloudy | visual | 0.644951 |  | reddish | visual | 0.804878 |
| cloying | haptic | 0.504582 |  | reddish | visual | 0.943299 |
| cloying | gustatory | 0.461538 |  | resounding | auditory | 0.614089 |
| coarse | haptic | 0.418478 |  | resounding | auditory | 0.585714 |
| coarse | haptic | 0.485531 |  | reverberating | auditory | 0.731449 |
| coconutty | gustatory | 0.434053 |  | reverberating | auditory | 0.388672 |
| coconutty | olfactory | 0.501377 |  | rhythmic | auditory | 0.413462 |
| cold | gustatory | 0.356589 |  | rhythmic | auditory | 0.562682 |
| cold | haptic | 0.42539 |  | ripe | gustatory | 0.342803 |
| colorful | visual | 0.945833 |  | ripe | gustatory | 0.279035 |
| colorful | visual | 0.922414 |  | rippled | visual | 0.382423 |
| colossal | visual | 0.734483 |  | rippled | visual | 0.436782 |
| colossal | visual | 0.536649 |  | roaring | auditory | 0.526923 |
| compact | visual | 0.558824 |  | roaring | auditory | 0.540404 |
| compact | haptic | 0.482972 |  | roasted | visual | 0.20205 |
| conical | visual | 0.432304 |  | roasted | olfactory | 0.2411 |
| conical | visual | 0.590747 |  | rotten | visual | 0.267564 |
| contoured | visual | 0.414948 |  | rotten | olfactory | 0.321859 |
| contoured | visual | 0.473239 |  | rough | visual | 0.616352 |
| cooing | auditory | 0.47027 |  | rough | haptic | 0.444976 |
| cooing | auditory | 0.573431 |  | round | visual | 0.482436 |
| cool | haptic | 0.440299 |  | round | visual | 0.719665 |
| cool | haptic | 0.382766 |  | rubbery | gustatory | 0.325188 |
| crackling | auditory | 0.336576 |  | rubbery | haptic | 0.377454 |
| crackling | auditory | 0.321839 |  | rumbling | auditory | 0.49589 |
| craggy | visual | 0.522818 |  | rumbling | auditory | 0.458904 |
| craggy | visual | 0.571429 |  | rustling | auditory | 0.373303 |
| crashing | visual | 0.459135 |  | rustling | auditory | 0.431034 |
| crashing | visual | 0.302655 |  | rusty | visual | 0.395876 |
| creaking | auditory | 0.491443 |  | rusty | visual | 0.364238 |
| creaking | auditory | 0.529399 |  | salty | gustatory | 0.465854 |
| creamy | haptic | 0.396588 |  | salty | gustatory | 0.418803 |
| creamy | gustatory | 0.371595 |  | savory | gustatory | 0.332779 |
| creased | visual | 0.425882 |  | savory | gustatory | 0.370927 |
| creased | visual | 0.495775 |  | scaly | haptic | 0.366013 |
| crimson | visual | 0.580716 |  | scaly | haptic | 0.465218 |
| crimson | visual | 0.855183 |  | scented | olfactory | 0.662252 |
| crinkled | visual | 0.450739 |  | scented | olfactory | 0.44802 |
| crinkled | visual | 0.326568 |  | scratchy | auditory | 0.502591 |
| crisp | olfactory | 0.278676 |  | scratchy | haptic | 0.515625 |
| crisp | gustatory | 0.197514 |  | scrawny | visual | 0.614907 |
| crooked | visual | 0.557971 |  | scrawny | visual | 0.539945 |
| crooked | visual | 0.55914 |  | screaming | auditory | 0.517966 |
| crowded | visual | 0.34965 |  | screaming | auditory | 0.58011 |
| crowded | visual | 0.347584 |  | screeching | auditory | 0.637462 |
| crunching | auditory | 0.252087 |  | screeching | auditory | 0.531328 |
| crunching | auditory | 0.425968 |  | shadowy | visual | 0.787149 |
| crying | auditory | 0.419831 |  | shadowy | visual | 0.776423 |
| crying | visual | 0.417391 |  | shaggy | visual | 0.5 |
| curly | visual | 0.363758 |  | shaggy | haptic | 0.455422 |
| curly | visual | 0.525381 |  | shallow | auditory | 0.544025 |
| curved | visual | 0.492537 |  | shallow | visual | 0.435768 |
| curved | visual | 0.521303 |  | sharp | gustatory | 0.415217 |
| cute | visual | 0.428899 |  | sharp | haptic | 0.509822 |
| cute | visual | 0.442516 |  | sheer | visual | 0.690377 |
| damp | haptic | 0.370098 |  | sheer | visual | 0.504505 |
| damp | haptic | 0.353783 |  | shimmering | visual | 0.533569 |
| dank | visual | 0.336538 |  | shimmering | visual | 0.677419 |
| dank | visual | 0.346864 |  | shiny | visual | 0.651163 |
| dappled | visual | 0.699422 |  | shiny | visual | 0.64557 |
| dappled | visual | 0.736909 |  | short | visual | 0.553977 |
| dark | visual | 0.405405 |  | short | visual | 0.558333 |
| dark | visual | 0.753623 |  | shrieking | auditory | 0.542284 |
| dazzling | visual | 0.911628 |  | shrieking | auditory | 0.528967 |
| dazzling | visual | 0.505319 |  | shrill | auditory | 0.462766 |
| dead | visual | 0.383795 |  | shrill | auditory | 0.829787 |
| dead | auditory | 0.629758 |  | silky | haptic | 0.516971 |
| deafening | auditory | 0.571429 |  | silky | haptic | 0.516854 |
| deafening | auditory | 0.722222 |  | silver | visual | 0.741935 |
| deep | visual | 0.930036 |  | silver | visual | 0.439678 |
| deep | visual | 0.471883 |  | sizzling | auditory | 0.236755 |
| delicious | gustatory | 0.354724 |  | sizzling | visual | 0.106354 |
| delicious | olfactory | 0.657439 |  | skinny | visual | 0.577205 |
| dim | visual | 0.254613 |  | skinny | visual | 0.504878 |
| dim | visual | 0.936937 |  | slick | visual | 0.446512 |
| dirty | visual | 0.32037 |  | slick | visual | 0.445652 |
| dirty | visual | 0.373757 |  | slimy | haptic | 0.341719 |
| downy | visual | 0.417625 |  | slimy | haptic | 0.380631 |
| downy | visual | 0.462484 |  | slippery | haptic | 0.467933 |
| drab | visual | 0.587333 |  | slippery | haptic | 0.36699 |
| drab | visual | 0.504801 |  | slushy | gustatory | 0.262579 |
| dry | gustatory | 0.364641 |  | slushy | haptic | 0.320463 |
| dry | visual | 0.410138 |  | small | visual | 0.474304 |
| dull | visual | 0.779592 |  | small | visual | 0.550279 |
| dull | auditory | 0.556391 |  | smelly | olfactory | 0.517073 |
| dusty | visual | 0.314928 |  | smelly | olfactory | 0.538058 |
| dusty | visual | 0.441805 |  | smoky | olfactory | 0.222034 |
| earthy | visual | 0.568643 |  | smoky | visual | 0.316629 |
| earthy | gustatory | 0.283019 |  | smooth | haptic | 0.451613 |
| echoing | auditory | 0.649231 |  | smooth | haptic | 0.497423 |
| echoing | auditory | 0.789343 |  | snarling | auditory | 0.660156 |
| eggy | olfactory | 0.523179 |  | snarling | auditory | 0.48329 |
| eggy | olfactory | 0.288889 |  | snorting | auditory | 0.544833 |
| elastic | haptic | 0.496124 |  | snorting | auditory | 0.63522 |
| elastic | haptic | 0.407407 |  | soapy | gustatory | 0.343669 |
| elegant | visual | 0.574286 |  | soapy | visual | 0.34749 |
| elegant | visual | 0.57377 |  | sodden | haptic | 0.36039 |
| empty | visual | 0.378906 |  | sodden | visual | 0.339779 |
| empty | visual | 0.32906 |  | soft | haptic | 0.436508 |
| enormous | visual | 0.476551 |  | soft | auditory | 0.821012 |
| enormous | visual | 0.540682 |  | solid | haptic | 0.403587 |
| faint | visual | 0.290323 |  | solid | haptic | 0.402273 |
| faint | olfactory | 0.5 |  | sonorous | auditory | 0.654711 |
| falling | visual | 0.735409 |  | sonorous | auditory | 0.741007 |
| falling | visual | 0.204412 |  | sore | haptic | 0.871245 |
| fat | visual | 0.503817 |  | sore | haptic | 0.401114 |
| fat | visual | 0.27707 |  | soundless | visual | 0.464497 |
| fatty | gustatory | 0.343612 |  | soundless | auditory | 0.569721 |
| fatty | visual | 0.346392 |  | sour | gustatory | 0.556507 |
| fetid | olfactory | 0.374724 |  | sour | gustatory | 0.506527 |
| fetid | olfactory | 0.324503 |  | sparkly | visual | 0.946188 |
| feverish | visual | 0.828704 |  | sparkly | visual | 0.31064 |
| feverish | haptic | 0.690236 |  | speckled | visual | 0.468708 |
| filthy | visual | 0.371429 |  | speckled | visual | 0.73454 |
| filthy | visual | 0.321755 |  | spicy | olfactory | 0.573668 |
| flaky | visual | 0.268293 |  | spicy | gustatory | 0.42887 |
| flaky | visual | 0.351579 |  | spiky | haptic | 0.479381 |
| flat | visual | 0.467626 |  | spiky | visual | 0.453581 |
| flat | gustatory | 0.39905 |  | spotted | visual | 0.92891 |
| fleshy | visual | 0.451087 |  | spotted | visual | 0.536818 |
| fleshy | gustatory | 0.289466 |  | square | visual | 0.538462 |
| flexible | haptic | 0.474453 |  | square | visual | 0.552279 |
| flexible | haptic | 0.503817 |  | squeaking | auditory | 0.5225 |
| flickering | visual | 0.654362 |  | squeaking | auditory | 0.70949 |
| flickering | visual | 0.808163 |  | squealing | auditory | 0.480952 |
| floppy | visual | 0.567935 |  | squealing | auditory | 0.589385 |
| floppy | haptic | 0.29264 |  | stagnant | olfactory | 0.273871 |
| floral | olfactory | 0.599315 |  | stagnant | visual | 0.475374 |
| floral | visual | 0.821277 |  | stale | gustatory | 0.46438 |
| flowery | visual | 0.549723 |  | stale | gustatory | 0.30767 |
| flowery | olfactory | 0.755396 |  | steep | visual | 0.620579 |
| fluffy | haptic | 0.471526 |  | steep | visual | 0.529101 |
| fluffy | visual | 0.284483 |  | stenchy | olfactory | 0.554572 |
| foamy | visual | 0.32906 |  | stenchy | olfactory | 0.477747 |
| foamy | visual | 0.360417 |  | sticky | haptic | 0.392996 |
| foggy | visual | 0.654362 |  | sticky | haptic | 0.454756 |
| foggy | visual | 0.487047 |  | stinging | haptic | 0.484375 |
| forked | visual | 0.644689 |  | stinging | haptic | 0.498592 |
| forked | visual | 0.48164 |  | stinky | olfactory | 0.383929 |
| fragrant | olfactory | 0.577844 |  | stinky | olfactory | 0.609091 |
| fragrant | olfactory | 0.560773 |  | straight | visual | 0.776471 |
| freezing | haptic | 0.363458 |  | straight | visual | 0.77381 |
| freezing | haptic | 0.420779 |  | striped | visual | 0.875648 |
| fresh | olfactory | 0.486486 |  | striped | visual | 0.959459 |
| fresh | olfactory | 0.165505 |  | strong | haptic | 0.497297 |
| frosty | visual | 0.434144 |  | strong | gustatory | 0.482838 |
| frosty | visual | 0.331839 |  | sturdy | visual | 0.50411 |
| fruity | olfactory | 0.471591 |  | sturdy | haptic | 0.468665 |
| fruity | gustatory | 0.422658 |  | sunny | visual | 0.482234 |
| fuzzy | haptic | 0.462075 |  | sunny | visual | 0.578947 |
| fuzzy | visual | 0.95045 |  | sweaty | visual | 0.29202 |
| gamy | gustatory | 0.247573 |  | sweaty | haptic | 0.278195 |
| gamy | gustatory | 0.3361 |  | sweet | olfactory | 0.696768 |
| garlicky | olfactory | 0.630573 |  | sweet | gustatory | 0.465066 |
| garlicky | gustatory | 0.404711 |  | swift | visual | 0.602305 |
| gigantic | visual | 0.366089 |  | swift | visual | 0.579832 |
| gigantic | visual | 0.509615 |  | swinging | visual | 0.463104 |
| giggling | auditory | 0.421171 |  | swinging | auditory | 0.656667 |
| giggling | auditory | 0.466825 |  | tall | visual | 0.620991 |
| glamorous | visual | 0.571288 |  | tall | visual | 0.808765 |
| glamorous | visual | 0.308285 |  | tangerine | visual | 0.769912 |
| glistening | visual | 0.889831 |  | tangerine | visual | 0.361478 |
| glistening | visual | 0.537433 |  | tangy | gustatory | 0.477064 |
| glittery | visual | 0.715356 |  | tangy | gustatory | 0.475128 |
| glittery | visual | 0.621019 |  | tapering | visual | 0.331461 |
| glossy | visual | 0.4725 |  | tapering | visual | 0.544118 |
| glossy | visual | 0.385776 |  | tarry | visual | 0.32636 |
| glowing | visual | 0.655455 |  | tarry | visual | 0.416021 |
| glowing | visual | 0.820084 |  | tart | gustatory | 0.474201 |
| gold | visual | 0.609907 |  | tart | gustatory | 0.508929 |
| gold | visual | 0.682862 |  | tasteless | visual | 0.421725 |
| gooey | haptic | 0.330709 |  | tasteless | gustatory | 0.553314 |
| gooey | gustatory | 0.243655 |  | tender | haptic | 0.553009 |
| gorgeous | visual | 0.578171 |  | tender | gustatory | 0.353896 |
| gorgeous | auditory | 0.798354 |  | tepid | haptic | 0.461538 |
| grainy | visual | 0.712727 |  | tepid | gustatory | 0.365285 |
| grainy | haptic | 0.330258 |  | thorny | haptic | 0.473538 |
| granular | visual | 0.280353 |  | thorny | haptic | 0.488312 |
| granular | haptic | 0.321596 |  | thudding | auditory | 0.539474 |
| grassy | gustatory | 0.442029 |  | thudding | auditory | 0.411622 |
| grassy | visual | 0.402322 |  | thumping | auditory | 0.449883 |
| gray | visual | 0.955556 |  | thumping | haptic | 0.538482 |
| gray | visual | 0.917749 |  | ticklish | haptic | 0.607455 |
| greasy | gustatory | 0.26484 |  | ticklish | haptic | 0.58104 |
| greasy | haptic | 0.414188 |  | tight | haptic | 0.526455 |
| green | visual | 0.643963 |  | tight | haptic | 0.486874 |
| green | visual | 0.575301 |  | tinkling | auditory | 0.356796 |
| grinding | visual | 0.284 |  | tinkling | auditory | 0.408163 |
| grinding | auditory | 0.438725 |  | tiny | visual | 0.585434 |
| gritty | visual | 0.382022 |  | tiny | auditory | 0.819328 |
| gritty | haptic | 0.43377 |  | tough | haptic | 0.483791 |
| groaning | auditory | 0.571429 |  | tough | gustatory | 0.361419 |
| groaning | auditory | 0.567568 |  | translucent | visual | 0.706678 |
| grotesque | visual | 0.621795 |  | translucent | visual | 0.854251 |
| grotesque | visual | 0.508108 |  | transparent | visual | 0.814672 |
| growling | auditory | 0.479218 |  | transparent | visual | 0.834008 |
| growling | auditory | 0.632968 |  | triangular | visual | 0.621455 |
| gurgling | auditory | 0.460396 |  | triangular | visual | 0.42723 |
| gurgling | auditory | 0.346792 |  | ugly | visual | 0.598071 |
| hairy | haptic | 0.414416 |  | ugly | visual | 0.533791 |
| hairy | visual | 0.5025 |  | uneven | visual | 0.654545 |
| handsome | visual | 0.679739 |  | uneven | haptic | 0.456311 |
| handsome | visual | 0.613095 |  | unripe | gustatory | 0.277448 |
| happy | visual | 0.589286 |  | unripe | gustatory | 0.34188 |
| happy | auditory | 0.712687 |  | vinegary | olfactory | 0.353333 |
| hard | gustatory | 0.407328 |  | vinegary | gustatory | 0.486413 |
| hard | haptic | 0.433708 |  | vivid | visual | 0.901408 |
| harsh | auditory | 0.521909 |  | vivid | visual | 0.281095 |
| harsh | haptic | 0.336207 |  | wailing | auditory | 0.494186 |
| heavy | visual | 0.474801 |  | wailing | auditory | 0.633333 |
| heavy | visual | 0.428635 |  | warbling | auditory | 0.706806 |
| herby | gustatory | 0.422222 |  | warbling | auditory | 0.572368 |
| herby | visual | 0.330693 |  | warm | haptic | 0.429671 |
| high | auditory | 0.791165 |  | warm | haptic | 0.557065 |
| high | visual | 0.758364 |  | waxy | haptic | 0.432373 |
| hissing | auditory | 0.5 |  | waxy | haptic | 0.439589 |
| hissing | auditory | 0.483627 |  | weak | haptic | 0.562937 |
| hoarse | auditory | 0.709821 |  | weak | visual | 0.864035 |
| hoarse | auditory | 0.459732 |  | weightless | visual | 0.672535 |
| hollow | visual | 0.4197 |  | weightless | haptic | 0.491573 |
| hollow | visual | 0.54388 |  | wet | visual | 0.343685 |
| honeyed | gustatory | 0.342056 |  | wet | haptic | 0.327451 |
| honeyed | auditory | 0.773333 |  | whining | auditory | 0.568915 |
| hot | gustatory | 0.38758 |  | whining | auditory | 0.502347 |
| hot | haptic | 0.366667 |  | whistling | auditory | 0.561497 |
| howling | auditory | 0.488943 |  | whistling | auditory | 0.578804 |
| howling | auditory | 0.548649 |  | white | visual | 0.788 |
| huge | visual | 0.382269 |  | white | visual | 0.889868 |
| huge | visual | 0.562147 |  | wide | visual | 0.540616 |
| humid | haptic | 0.349765 |  | wide | visual | 0.666667 |
| humid | haptic | 0.307571 |  | wiry | visual | 0.546125 |
| humming | auditory | 0.603499 |  | wiry | haptic | 0.436474 |
| humming | auditory | 0.755304 |  | wispy | visual | 0.792579 |
| hushed | auditory | 0.529915 |  | wispy | visual | 0.416058 |
| hushed | auditory | 0.615385 |  | woolly | haptic | 0.517857 |
| husky | haptic | 0.385765 |  | woolly | haptic | 0.486811 |
| husky | auditory | 0.889831 |  | yellow | visual | 0.816794 |
| icy | haptic | 0.563025 |  | yellow | visual | 0.849206 |

List of 387 Adjectives and their Sensory Values for each corresponding Representational System, which across both of the modalities equals 774 words.
