# Supplementary material for: Using Shakespeare's Sotto Voce to Determine True Identity From Text
Source: Front Psychol. 2018 Mar 15;9:289. doi: 10.3389/fpsyg.2018.00289 (PMC5862847; doi:10.3389/fpsyg.2018.00289)

Additional External Data

Table 1 Sensory Correlation Matrix

| **Correlation Matrix^a^** | | | | | | | | | |
| --- | --- | --- | --- | --- | --- | --- | --- | --- | --- |
|  |  | Richness | P_Pronouns | RA Score | Auditory | Gustatory | Haptic | Olfactory | Visual |
| Correlation | Richness | 1.000 | .399 | -.833 | .606 | .311 | .195 | .296 | .372 |
|  | P_Pronouns | .399 | 1.000 | -.451 | .252 | .210 | .340 | .169 | .362 |
|  | RA Score | -.833 | -.451 | 1.000 | -.439 | -.430 | -.343 | -.430 | -.520 |
|  | Auditory | .606 | .252 | -.439 | 1.000 | -.119 | -.038 | .167 | .284 |
|  | Gustatory | .311 | .210 | -.430 | -.119 | 1.000 | .772 | .628 | .571 |
|  | Haptic | .195 | .340 | -.343 | -.038 | .772 | 1.000 | .632 | .715 |
|  | Olfactory | .296 | .169 | -.430 | .167 | .628 | .632 | 1.000 | .784 |
|  | Visual | .372 | .362 | -.520 | .284 | .571 | .715 | .784 | 1.000 |
| Sig. (1-tailed) | Richness |  | .001 | .000 | .000 | .009 | .073 | .013 | .002 |
|  | P_Pronouns | .001 |  | .000 | .029 | .058 | .005 | .104 | .003 |
|  | RA Score | .000 | .000 |  | .000 | .000 | .005 | .000 | .000 |
|  | Auditory | .000 | .029 | .000 |  | .189 | .389 | .107 | .016 |
|  | Gustatory | .009 | .058 | .000 | .189 |  | .000 | .000 | .000 |
|  | Haptic | .073 | .005 | .005 | .389 | .000 |  | .000 | .000 |
|  | Olfactory | .013 | .104 | .000 | .107 | .000 | .000 |  | .000 |
|  | Visual | .002 | .003 | .000 | .016 | .000 | .000 | .000 |  |
| a. Determinant = .004 | | | | | | | | | |

Table 2 Sensory KMO and Bartlett's Test

| **KMO and Bartlett's Test** | | |
| --- | --- | --- |
| Kaiser-Meyer-Olkin Measure of Sampling Adequacy. | | .722 |
| Bartlett's Test of Sphericity | Approx. Chi-Square | 290.851 |
|  | df | 28 |
|  | Sig. | .000 |

Table 3 Sensory Communalities Table

| **Communalities** | | |
| --- | --- | --- |
|  | Initial | Extraction |
| Richness | 1.000 | .832 |
| Rel_Gender_S896 | 1.000 | .354 |
| RA Score | 1.000 | .787 |
| Auditory | 1.000 | .695 |
| Gustatory | 1.000 | .772 |
| Haptic | 1.000 | .830 |
| Olfactory | 1.000 | .711 |
| Visual | 1.000 | .770 |
| Extraction Method: Principal Component Analysis. | | |

Table 4 Sensory Total Variance Explained

| **Total Variance Explained** | | | | | | | | | |
| --- | --- | --- | --- | --- | --- | --- | --- | --- | --- |
| Component | Initial Eigenvalues | | | Extraction Sums of Squared Loadings | | | Rotation Sums of Squared Loadings | | |
|  | Total | % of Variance | Cumulative % | Total | % of Variance | Cumulative % | Total | % of Variance | Cumulative % |
| 1 | 3.965 | 49.565 | 49.565 | 3.965 | 49.565 | 49.565 | 3.163 | 39.537 | 39.537 |
| 2 | 1.786 | 22.322 | 71.887 | 1.786 | 22.322 | 71.887 | 2.588 | 32.350 | 71.887 |
| 3 | .807 | 10.093 | 81.980 |  |  |  |  |  |  |
| 4 | .652 | 8.148 | 90.128 |  |  |  |  |  |  |
| 5 | .327 | 4.084 | 94.213 |  |  |  |  |  |  |
| 6 | .221 | 2.766 | 96.979 |  |  |  |  |  |  |
| 7 | .126 | 1.581 | 98.560 |  |  |  |  |  |  |
| 8 | .115 | 1.440 | 100.000 |  |  |  |  |  |  |
| Extraction Method: Principal Component Analysis. | | | | | | | | | |


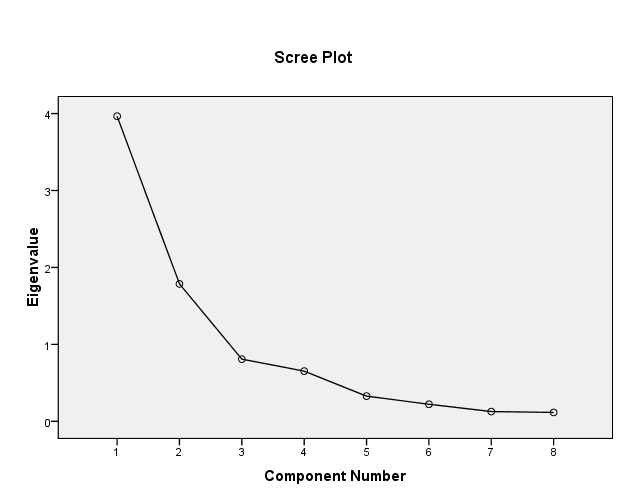


Figure 1 Scree plot of 8 factors

Table 5 Sensory Component matrix

| **Component Matrix^a^** | | |
| --- | --- | --- |
|  | Component | |
|  | 1 | 2 |
| Richness | .684 | -.603 |
| Rel_Gender_S896 | .532 |  |
| RA Score | -.787 | .409 |
| Auditory | .388 | -.737 |
| Gustatory | .734 | .483 |
| Haptic | .755 | .510 |
| Olfactory | .777 | .327 |
| Visual | .855 |  |
| Extraction Method: Principal Component Analysis. | | |
| a. 2 components extracted. | | |

**Summary of LDA Canonical Discriminant Functions**

Table 6: LDA Eigenvalues of the first two canonical functions

| **Eigenvalues** | | | | |
| --- | --- | --- | --- | --- |
| Function | Eigenvalue | % of Variance | Cumulative % | Canonical Correlation |
| 1 | 2.266^a^ | 79.1 | 79.1 | .833 |
| 2 | .598^a^ | 20.9 | 100.0 | .612 |
| a. First 2 canonical discriminant functions were used in the analysis. | | | | |

Table 7: LDA Wilks' Lambda results of the two canonical functions

| **Wilks' Lambda** | | | | |
| --- | --- | --- | --- | --- |
| Test of Function(s) | Wilks' Lambda | Chi-square | df | Sig. |
| 1 through 2 | .192 | 66.919 | 16 | .000 |
| 2 | .626 | 18.985 | 7 | .008 |

Table 8: LDA Discriminant Function coefficients for the two canonical functions

| **Standardized Canonical Discriminant Function Coefficients** | | |
| --- | --- | --- |
|  | Function | |
|  | 1 | 2 |
| Richness | 1.115 | .092 |
| Gender | .369 | -.346 |
| RA Score | -.119 | .832 |
| Auditory | .816 | .394 |
| Gustatory | -.084 | .672 |
| Haptic | -1.733 | -.127 |
| Olfactory | .642 | -.251 |
| Visual | -.180 | .831 |

Table 9: LDA Group Centroids of the three Playwrights for both canonical functions

| **Functions at Group Centroids** | | |
| --- | --- | --- |
| Playwrights | Function | |
|  | 1 | 2 |
| 1 | -.645 | .070 |
| 2 | 3.423 | .327 |
| 3 | 1.205 | -5.037 |
| Unstandardized canonical discriminant functions evaluated at group means | | |

Figure 2: The first two dimensions of a canonical discriminant analysis applied to the uncontested works of Shakespeare, Marlowe and Cary


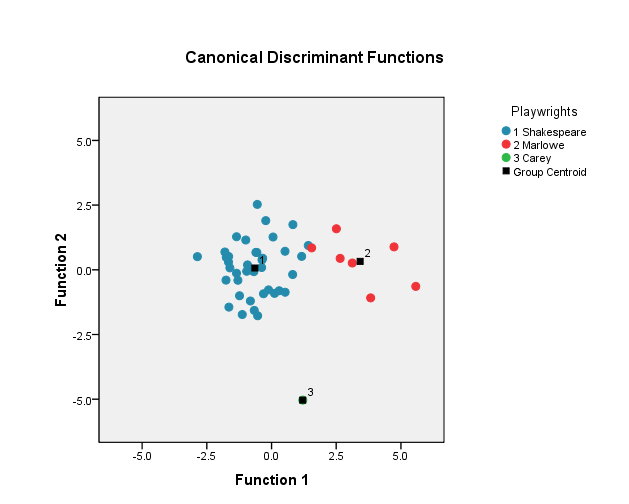

Supplement: Supplementary file 5 [file DataSheet1.DOCX]
